# Targeting the integrated stress response or Ataxin-2 alleviates neurodegeneration in PolyGR models of C9orf72 associated frontotemporal dementia and amyotrophic lateral sclerosis

**DOI:** 10.1186/s40478-026-02301-2

**Published:** 2026-05-05

**Authors:** Nikki S. Harper, Joanne L. Sharpe, Jasmine Speranza, Ravinder Gulia, Jeffrey X. Chen, Scott P. Allen, Manpreet S. Atwal, Stuart Pickering-Brown, Matthew R. Livesey, Craig L. Bennett, Andreas Prokop, Albert R. La Spada, Ryan J. H. West

**Affiliations:** 1 Division of Neuroscience and Experimental Psychology, Faculty of Biology, Medicine and Health, University of Manchester, Manchester, UK; 2 Sheffield Institute for Translational Neuroscience (SITraN), University of Sheffield, 385a Glossop Road, Sheffield, S10 2HQ UK; 3 Neuroscience Institute, University of Sheffield, Sheffield, S10 2TN UK; 4 Department of Pathology and Laboratory Medicine, University of California, Irvine, Irvine, CA 92617 USA; 5 UCI Center for Neurotherapeutics, University of California, Irvine, Irvine, CA 92697 USA; 6 Division of Molecular and Cellular Function, Faculty of Biology, Medicine and Health, The University of Manchester, Manchester, UK; 7 Department of Biological Chemistry, University of California, Irvine, Irvine, CA 92617 USA; 8 Department of Neurology, University of California, Irvine, Irvine, CA 92617 USA; 9 Department of Neurobiology and Behavior, University of California, Irvine, Irvine, CA 92697 USA

**Keywords:** C9orf72, Frontotemporal dementia, Ataxin-2, Amyotrophic lateral sclerosis, Motor neurone disease, Integrated stress response, Stress granules, *Drosophila*

## Abstract

Frontotemporal dementia (FTD) and amyotrophic lateral sclerosis (ALS) are fatal, early-onset neurodegenerative diseases. The most common genetic cause of FTD and ALS is a G4C2 hexanucleotide repeat expansion in the *C9orf72* gene. This mutation leads to the production of toxic dipeptide repeat proteins (DPRs), via repeat-associated non-AUG (RAN) translation. These DPRs disrupt stress granule (SG) dynamics, with SG regulators such as Ataxin-2 (ATXN2) implicated in disease risk. The integrated stress response (ISR), a key driver of SG formation via eIF2α phosphorylation, has been linked to *C9orf72* expansions, but the role of individual DPRs in ISR activation remains unclear. Here, using *Drosophila* models expressing physiologically relevant repeat length DPRs, we identify poly(GR) as a novel activator of the ISR, inducing early and sustained eIF2α phosphorylation and SG accumulation prior to motor decline. Genetic inhibition of the ISR or knockdown of *ATX2*, the *Drosophila* orthologue of *ATXN2*, rescues motor deficits in these models. *ATXN2* knockdown also reduces poly(GR) toxicity in mouse primary neurons. These findings position poly(GR) as a key driver of ISR activation and highlight *ATXN2* and the ISR as promising therapeutic targets in *C9orf72*-associated FTD/ALS.

**Supplementary Information:**

The online version contains supplementary material available at 10.1186/s40478-026-02301-2.

## Introduction

Frontotemporal dementia (FTD) and amyotrophic lateral sclerosis (ALS) are two devastating early onset neurodegenerative diseases, for which there is currently no cure. FTD is characterised by the progressive degeneration of the frontal and temporal lobes of the brain leading to severe behavioural and language impairments. In contrast, ALS is hallmarked by upper and lower motor neuron loss resulting in debilitating motor deficits. The two conditions share a clinical, pathological and genetic spectrum, with the most common genetic cause of both FTD and ALS being a hexanucleotide (G_4_C_2_) repeat expansion mutation within the *C9orf72* gene [12, 35]. Both loss-of-function, through C9orf72 haploinsufficiency, and gain-of-function mechanisms have been proposed to explain how C9orf72 expansions contribute to disease. However, it is the bidirectional repeat-associated non-AUG (RAN) translation of sense and antisense C9orf72 repeat RNA, facilitating the synthesis of five dipeptide repeat proteins (DPRs), that is thought to be the most prominent driver of neurodegeneration in C9orf72-FTD/ALS [5, 30, 48]. The five DPRs comprise poly-proline-alanine (AP), poly-proline-arginine (PR), poly-glycine-arginine (GR), poly-glycine-alanine (GA) and poly-glycine-proline (GP).

DPRs aggregate within neurons and have been implicated in the disruption of a range of cellular processes, including stress granule dynamics [25, 50]. The arginine-rich DPRs, GR and PR, have been shown to enhance the formation of stress granules, impair their disassembly and form pathological aggregates that colocalise with, and promote the phase separation of, several stress granule-associated RNA-binding proteins. These include Tia1 cytotoxic granule-associated RNA binding protein (TIA1), G3BP Stress Granule Assembly Factor 1 (G3BP1) and Ataxin-2 (ATXN2) [7, 21, 25, 44, 50]. Stress granule formation occurs, in part, following phosphorylation and activation of eukaryotic initiation factor 2α (eIF2α). eIF2α is a core component of the integrated stress response (ISR), a regulatory pathway that maintains cellular homeostasis in response to physiological and pathological stress stimuli. Increasing evidence implicates both the ISR and dysregulated stress granule dynamics in the pathogenesis of ALS and FTD [10, 15, 18, 20, 32, 39, 49, 50]. C9orf72 expansions have been associated with activation of the ISR, with increased stress granule formation and eIF2α phosphorylation observed in C9orf72 patient-derived cell models and patient tissue [18, 20, 32, 39, 49, 50]. Previous work has also demonstrated that RAN translation of *C9orf72* repeat RNA requires an eIF2α phosphorylation-dependent alteration in start codon fidelity [10, 15]. This suggests that RAN translation, and therefore DPR synthesis, is selectively enhanced by activation of the ISR. If DPRs independently induce cellular stress, promote chronic activation of the ISR and enhance the aberrant formation and persistence of stress granules, this may in turn contribute to a potential positive feed-back loop, driving a neurotoxic cascade. Despite this, mechanisms leading to eIF2α phosphorylation, and whether it is underpinned by specific DPR species remains unclear.

In this study we have utilised our *Drosophila* models, which individually express each DPR at a length that is pathologically and physiologically comparable to the longest repeat lengths observed in patients (> 1000 repeats), to show that pan-neuronal expression of 1000-repeat GR promotes eIF2α phosphorylation and stress granule accumulation. eIF2α phosphorylation occurs early, preceding motor decline, suggesting a sustained and chronic activation of the ISR. Furthermore, we show that targeted inhibition of the ISR or knockdown of the *Drosophila* ATXN2 orthologue, *ATX2*, a key regulator of stress granule formation, is sufficient to rescue motor dysfunction in these models. Finally, we reveal knockdown of *ATXN2* is sufficient to reduce toxicity in mouse primary neurons transduced with PolyGR. These findings reveal ATXN2, and the ISR, to be potential therapeutic targets for the treatment of FTD and ALS associated with C9orf72 hexanucleotide expansions.

## Methods

### *Drosophila* stocks and maintenance

Unless stated otherwise *Drosophila* were raised on standard cornmeal–yeast medium (7.2% w/v maize flour, 5% w/v yeast, 8% w/v glucose, 0.8% w/v agar) at 25 °C on a 12 h light: dark cycle. UAS-AP(1024)-eGFP (Flybase ID FBti0213155), UAS-PR(1100)-eGFP (Flybase ID Fbti0213157), UAS-GA(1020)-eGFP (Flybase ID Fbti0213158), and UAS-GR(1136)-eGFP (Flybase ID Fbti0213156), referred to throughout as 1000 repeat lines, were described previously [48]. nSyb-Gal4 (II) was described previously [48]. GMR-Gal4 (RRID: BDSC_9146), UAS-mCD8-GFP (RRID: BDSC_32184), UAS-PPP1R15 (RRID: BDSC_76250), UAS-mCherry-RNAi (RRID: BDSC_35785), UAS-ATX2-RNAi^2^ (ATX2^HMS02726^, RRID: BDSC_44012), UAS-ATXN2.22Q (RRID: BDSC_79594), UAS-ATXN2.32Q (RRID: BDSC_79593), UAS-GCN2-RNAi^1^ (GCN2^HMC06316^, RRID: BDSC_67215), UAS-PEK-RNAi (PEK^HMJ02063^, RRID: BDSC_42499) and PEK^e01744^ (RRID: BDSC_85557) were obtained from the Bloomington *Drosophila* Stock Center (BDSC). UAS-ATX2 (FlyORF: F001031) was obtained from the FlyORF Zurich ORFeome stock center [6]. UAS-ATX2-RNAi^1^ (ATX2^GD11562^, VDRC ID: 34955) and UAS-GCN2-RNAi^2^ (GCN2^GD9162^, VDRC ID: 32664) were obtained from the Vienna *Drosophila* Resource Center (VDRC). Wild-types are Canton S outcrossed to *w*^*1118*^. All experiments were performed using flies from at least 3 independent crosses.

### SDS-PAGE and western blotting

Flies pan-neuronally (nSyb-Gal4) expressing either UAS-AP(1000), UAS-GA(1000), UAS-PR(1000), UAS-GR(1000) or UAS-mCD8-GFP controls were snap frozen on dry ice and heads removed by vortexing, as described previously [41]. 20–40 heads, per genotype, per repeat, were lysed in RIPA buffer (10 mM Tris–Cl pH 8.0, 1 mM EDTA, 0.5 mM EGTA, 1% Triton X-100, 0.1% Sodium deoxycholate, 0.1%SDS, 140 mM NaCl) containing Halt™ Protease and Phosphatase Inhibitor Cocktail, EDTA-free (100X) (Thermo Fisher #78441). Lysates were cleared by centrifugation (15-minutes 13,000 rpm), concentrations quantified using A Pierce™ BCA Protein Assay Kit (Thermo Fisher #23227) and diluted to equal concentrations. Lysates were mixed with laemmli buffer (20 µg total protein, 2% SDS, 10% glycerol, 60 mM Tris-CL pH 6.8, 0.01% bromophenol blue, 20 mM DTT) and run on 12% SDS-PAGE gels. Transfers were performed for 1 h (100 V, 20% Methanol, Hybond-P polyvinylidene difluoride (PVDF) membrane (Amersham #RNP1416F)). Primary antibodies used were anti-EIF2S1 (Abcam Cat# ab26197, RRID: AB_2096478, Rabbit, 1:1000), anti-EIF2S1 (phospho S51) (Abcam Cat# ab32157, RRID: AB_732117, Rabbit, 1:1000), anti-ATX2 (Chicken, 1:1000, A gift from Prof. Mani Ramaswami, Trinity College, Dublin [2]), anti-α-tubulin (Sigma-Aldrich Cat# T9026, RRID: AB_477593, Mouse, 1:4000) and anti-beta-tubulin (DSHB Cat# E7, RRID: AB_528499, mouse, 1:5000). All primary antibody incubations were performed at 4 °C overnight. Secondary antibodies were goat anti-rabbit-HRP (Agilent Cat# P0448, RRID: AB_2617138, 1:1000), goat anti-mouse-HRP (Agilent Cat# P0447, RRID: AB_2617137, 1:1000), goat anti-chicken-HRP (Jackson ImmunoResearch Labs Cat# 103-035-155, RRID: AB_2337381, 1:25,000), donkey anti-rabbit Alexa Fluor 790 (Jackson ImmunoResearch abs Cat# 711-655-152, AB_2340628, 1:25,000) and donkey anti-mouse 800CW (LICORbio Cat# 926-32212, RRID: AB_621847, 1:25,000). Secondary antibody incubations were performed for 1 h at room temperature. Detection was performed using enhanced chemiluminescence substrate (ECL, Promega W1015) imaged on a G: box imaging unit (Syngene) with ECL settings or using a LICORbio Osyssey Classic Imager. Western blots were quantified using ImageJ.

### Negative geotaxis motor assays

Without anaesthetisation, 5–10 male flies were placed in glass boiling tubes, mounted onto a custom-made apparatus with a white background. After acclimatisation, the apparatus was banged down three times in a consistent manner, causing the flies to elicit a startle-induced negative geotaxis escape response. A Konig Full HD Action camera recorded the flies as they climbed, for a maximum of 60 s, or until all flies reached the top of the tube. Videos were processed and analysed using ImageJ. Briefly, a custom macro was used to threshold the images, distinguishing each fly and excluding the background. The Mtrack2 plugin tracked the movement of individual flies between frames (30 frames per second). The relative position of each individual fly during a 15 s period was used to calculate the median climbing speed. Assays were carried out at 25 °C and at the same time each day, with a half-hour window, to mitigate the impact of circadian differences. For all assays, expression of constructs was driven using pan-neuronal nSyb-Gal4, on the second chromosome. 20–30 flies per genotype were assessed from a minimum of three independent crosses.

### Activity monitoring assays

The TriKinetics *Drosophila* Activity Monitor 10 (DAM10) system was used to measure the activity of *Drosophila*, by recording infra-red beam breaks caused by the movement of the fly. Anaesthetised flies were placed in transparent tubes, supplied with food and sealed with a rubber bung at one end and secured with cotton wool at the other. Up to 32 tubes were loaded into the monitor at any given time. The monitor was housed in an incubator, maintained at 25 °C with constant humidity and subject to 12-hour light dark cycles. Flies were given 24 h to acclimatise and recover from the CO_2_ anaesthetisation, before activity was recorded. The locomotor activity of *Drosophila* was measured over 24 h. The DAM10 TriKinetics software read at 30 s intervals across all four infra-red channels. A ‘move’ was registered each time a fly entered an infra-red beam after exiting another, preventing continuous movement around a single beam from being recorded. The DAMFileScan113 software was used to quantify the number of ‘moves’ in 24 hours.

### Genetic interaction eye screens

The GMR-Gal4 line was used to drive expression of the 1000 repeat UAS-DPR constructs and the UAS-Ataxin-2 constructs, within the fly eye. For genetic interaction experiments flies were maintained at 29 °C. Eye phenotypes were examined under a stereo microscope, within 3 days post-eclosion and imaged using a Leica MZ10F microscope with a Leica DFC410 C camera and Leica LAS software. Flies were taken from a minimum of 3 crosses per genotype, with at least 100 flies scored per genotype.

### Seizure assays

Mechanical induced seizure assays were performed as described previously [29]. Briefly ~ 5–6 flies were placed into an empty, transparent *Drosophila* food vial and vortexed using a Vortex-genie 2 lab vortex (Scientific Industries Inc) at the maximum speed setting (10) for 10 s. Immediately following vortexing, video recordings were made of flies until all had recovered. Videos were analysed for the presence or absence, and length of, seizures using ImageJ.

### *Drosophila* primary cell culture

Primary neurons were cultured following protocols described previously [33, 34, 37, 48]. Briefly, embryos were dechorinated with 50% bleach and selected at stage 11. Embryos were sterilised in 70% ethanol, then washed in Schneider’s medium, supplemented with 20% foetal bovine serum. Embryos were mechanically and chemically dispersed in dispersion medium (0.002 g Collagenase Type 1, 0.004 g Dispase II, Hank’s Balanced Salt Solution) and left to incubate for 5 min at 37 °C. After centrifugation, the pellet was washed and resuspended in Schneider’s medium, then plated onto concanavalin A (5 µg/ml) coated glass coverslips. Cells were grown as hanging drop cultures, using custom chambers, and incubated at 26 °C for 5 days in vitro. Primary neurons were fixed in 4% paraformaldehyde (PFA) in 0.05 M PBS (pH 7-7.2) for 30 min, then washed three times in PBS with 0.3% Triton X-100 (PBS-T) prior to antibody staining. Primary antibodies were anti-tubulin (clone YL 1/2, Sigma-Aldrich, RRID: AB_2890657, rat, 1:500), anti-dFMR1 (5A11, DSHB, RRID: AB_528252, mouse, 1:20), anti-GFP (Alexa Fluor 488-conjugated, ChromoTek, RRID AB_2827573, alpaca, 1:1000), anti-GFP (ab6556, abcam, RRID: AB_305564, rabbit, 1:500,), anti-HRP (Cy3 conjugated, Stratech, RRID AB_2338959, goat, 1:200) and anti-eIF4E (Santa Cruz Biotechnology Cat# sc-9976, RRID: AB_627502, mouse, 1:50). Secondary antibodies were Cy3 conjugated anti-Rat (Jackson ImmunoResearch, RRID: AB_2340667, donkey, 1:200), anti-Mouse Alexa Fluor 647 (Jackson ImmunoResearch, RRID: AB_2340863, donkey, 1:500) and FITC conjugated anti-Rabbit (Jackson ImmunoResearch, RRID: AB_2315776, donkey, 1:500). Coverslips were mounted on slides using ProLong Gold and imaged using an Olympus BX50WI microscope, at 100X magnification, using Carl Zeiss™ Immersol™ Immersion Oil (518 N). For sodium arsenite treatment coverslips containing the hanging-drop cultures were removed from the chamber slides on the day of fixation (day 5 in culture) and the remaining growth media removed from the chamber. Chambers were briefly washed with 50 µl 0.5 mM NaAsO_2_ (Thermo Fisher #AA41533AP) diluted in growth media. 50 µl of the 0.5 mM NaAsO_2_ diluted in growth media was then added to each chamber and the coverslip replaced on its respective slide. Slides were returned to 26 °C for 180 min before the coverslips were removed, washed and fixed in 4% paraformaldehyde (PFA) in 0.05 M PBS, as above.

### Brain dissection and immunohistochemistry

*Drosophila* adult brains were dissected in PBS (137 mM NaCl, 2.7 mM KCl, 10 mM Na2HPO4 and 1.8 mM KH2PO4 in dH2O) on a sylgard plate (Silicone elastromere kit, DowCorning, MI, USA) prior to fixation in 3.7% formaldehyde (FA, Sigma #252549) in PBS for 1 h. Following three washes in 0.5% PBS-T (0.5% triton X-100 in PBS (v/v)) brains were incubated in primary antibodies anti-dFMR1 (5A11, DSHB, RRID: AB_528252, mouse, 1:20) and anti-ATX2 (Chicken, 1:500, A gift from Prof. Mani Ramaswami, Trinity College, Dublin [2]) in 0.5% PBS-T overnight, at 4 °C, with rotation. After incubation, brains were washed three times in 0.5% PBS-T, then incubated with secondary antibodies anti-Chicken Alexa Fluor 647 (Jackson ImmunoResearch, RRID: AB_2340379, Donkey, 1:500) and Cy3-conjugated anti-mouse (Jackson ImmunoResearch, RRID: AB_2315777, Donkey, 1:500) in 0.5% PBS-T for 1 h at room temperature, in darkness, with rotation. Brains were washed three times in 0.5% PBS-T and mounted using Vectashield^®^ hard set mounting media (Vector Laboratories LTD, UK #H-1400-10) before imaging using either a Leica CTR6000 or EVOS M5000 widefield fluorescence microscope. Identical microscope settings were for all samples. Quantification of FMR1 and ATX2 puncta was performed in a define 5800 µm^2^ region of interest within the protocerebrum, imaged using either a 40× or 63× oil objective, using the same stack depth and slice-interval. Z-stack image were blinded, subjected to the background subtraction (rolling ball radius of 50 pixels) and a threshold applied equally prior to manual quantification. 3 individual regions of interest were quantified per brain and the mean taken. For heat stress experiments flies were placed at 37 °C for 2 hours before dissection.

### iPSC-derived motor neurons

Induced pluripotent stem cell (iPSC) lines CS28iALS-C9nxx (RRID: CVCL_W558) and CS29iALS-C9nxx (RRID: CVCL_W559), described and characterised previously, were obtained from Cedars-Sinai iPSC Biorepository [1, 9]. iPSCs were maintained at 37 °C and 5% CO_2_ in Matrigel-coated (Corning) 6-well plastic dishes in mTeSR™ medium (StemCell Technologies). Media was replaced every 2 days. iPSC to motor neuron differentiation was performed by adapting an established protocol [13]. iPSC lines at 100% confluency were switched to a default base media containing Knockout™ DMEM/F12 media (48% v/v, Gibco), Neurobasal (48% v/v, Gibco), B-27 supplement (1% v/v, Gibco), Glutamax™ (1% v/v, Gibco), N-2 supplement (0.5% v/v, Gibco), penicillin/streptomycin (1% v/v, Lonza). From 1 to 6 days the default media was supplemented with CHIR99021 (3 µM, Merck Millipore), DMH-1 (2 µM, Merck Millipore) and SB431542 (2 µM, Peprotech) to promote neural induction. On day 7, cultures were switched to a media, consisting of the former default media supplemented with the addition of retinoic acid (0.1 µM, StemCell Technologies) and purmorphamine (0.5 µM, Merck Millipore). Between day 7–9, cells were passaged using Accutase and replated onto Matrigel-coated 6-well plates. On day 12, NPCs were passaged, replated, and further maintained/expanded in the prior media additionally supplemented with valproic acid (0.5 µM, Merck Millipore). Media changes were performed every 24 h until the NPC expansion stage. At 100% confluence, NPCs were transitioned to motor neuron differentiation media, comprising the base media supplemented with retinoic acid (0.5 µM) and purmorphamine (0.1 µM). At day 19, cultures were maintained in the former media, but with further supplements of Compound-E (0.1 µM, StemCell Technologies), BDNF (10 ng/mL), CNTF (10 ng/mL, Peprotech) and IGF-1 (10 ng/mL, Peprotech). On days 20–21, the cultures were resuspended and replated onto Matrigel-coated 13 mm glass coverslips supplemented with Y27632 (10 µM). Fresh medium was added 24 h after replating and changed every 48 h thereafter. On day 29, cultures were maintained in a media comprising Neurobasal media, B-27 supplement (2% v/v), penicillin/Streptomycin (1% v/v), BDNF (10 ng/ml), CNTF (10 ng/ml) and IGF-1 (10 ng/ml) until experimental use. Cells were stained with anti-GR (Millipore, Cat# MABN778, RRID: AB_2728664, Rat 1:500), CoraLite Plus 488-conjugated anti-ATXN2 (Proteintech, Cat# CL488-68316, RRID: AB_3084472, Mouse 1:500) and CoraLite Plus 647-conjugated anti-G3BP1 (Proteintech, Cat# CL647-66486, RRID: AB_2935056, Mouse 1:500) overnight, at 4 °C. Secondary antibodies were anti-Rat (Jackson ImmunoResearch, RRID: AB_2340667, Donkey, 1:500) in 0.5% PBS-T for 1 h at room temperature, in darkness, with agitation. Coverslips were mounted in Vectashield^®^ mounting media with DAPI (Vector Laboratories LTD, UK #H-1200) and imaged using an Opera Phenix high-content imaging system. Colocalization analysis was performed using the Coloc2 plugin in Fiji. Following automatic thresholding using the Costes threshold regression, the degree of spatial co-occurrence and signal correlation were quantified using thresholded Manders’ overlap coefficients (tM1 and tM2) and thresholded Pearson’s correlation coefficient (R), respectively. To verify that the observed colocalization was statistically significant and not due to random spatial overlap, Costes randomization was performed using 100 iterations and a point spread function (PSF) estimate of 3 pixels.

### Mouse primary neurons

Primary cortical neurons (PCNs) were prepared from P0/P1 C57BL/6J mouse pups. Following euthanasia, cortices were dissected, and tissues were dissociated using an enzymatic solution containing papain for 30 min at 37 °C. Papain activity was then inactivated with serum-containing media. Cells were triturated with a pipette to obtain single-cell suspensions, and neurons plated on pre-coated coverslips (50 µg/mL poly-D-lysine + 0.1 M sodium borate, pH 8.4) at a density of ~ 70,000 cells per well in clear-bottom 96-well plates. Cultures were maintained in Neurobasal medium supplemented with 0.5 mM glutamine and B27. At day 3 Glial growth was inhibited by adding FUDR (floxuridine) to the culture. At day 6 lentivirus containing shRNA sequences for Scramble or Atxn2 (VB900142-8349skf) were added at a Multiplicity of Infection (MOI) of 5. At day 8 AAVs expressing GFP, GR100, and GR200 (generously provided by Leonard Petrucelli [11]) were applied at a MOI of 50,000. PCNs were fixed (Day 15) in 4% PFA, permeabilised with 0.1% Triton X-100 and incubated with anti-MAP2 polyclonal antibody (Thermo Fisher Scientific Cat# PA1-10005, RRID: AB_1076848, Chicken, 1:500) at 4 °C overnight. PCNs were washed with PBS and incubated with a secondary antibody (Thermo Fisher Scientific Cat# A32932, RRID: AB_2762844, Goat) for 1 h at room temperature. Finally, PCNs were stained with Hoechst (1:10,000 dilution, H1399, Fisher Scientific) and imaged using a Nikon AR confocal microscope (20× objective). Sholl analysis was performed using the Sholl plugin in Fiji [40].

## Results

### 1000-repeat polyGR leads to an increase in eIF2α phosphorylation in the *Drosophila* nervous system

**Fig. 1:**
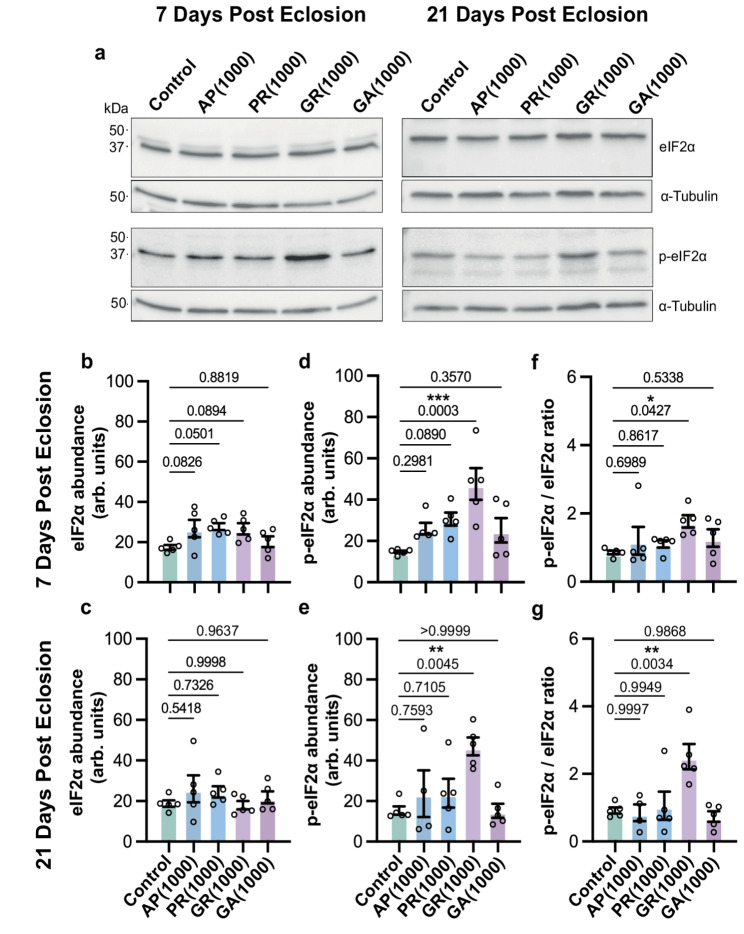
Pan-neuronal expression of GR(1000) increases eIF2α phosphorylation in *Drosophila* heads. **a** Representative western blots showing elevated levels of p-eIF2α in the heads of *Drosophila* pan-neuronally (nSyb-Gal4) expressing 1000-repeat DPRs, or an mCD8-GFP control. **b**–**g** Quantification of the relative abundance of eIF2α (**b**,** c**), p-eIF2α (**d**,** e**) and the p-eIF2α/eIF2α ratio (**f**,** g**) in the heads of flies pan-neuronally expressing 1000-repeat DPRs, or an mCD8-GFP control at 7 and 21 days post-eclosion. Error bars = SEM, ANOVA with post-hoc Dunnett’s comparison to controls * *p* < 0.05, ** *p* < 0.01, *** *p* < 0.001

Aberrantly increased phosphorylation of the ISR master regulator eIF2α has previously been observed in C9orf72 repeat expansion patient tissues, and patient-derived cell models [18, 20, 32, 39, 49, 50]. P-eIF2α dependent activation of the ISR has also been shown to promote RAN translation, leading to increased DPR production, which may in turn activate the ISR in a pathological feedback loop [16]. We hypothesise this leads to chronic activation of the ISR, which has previously been shown to cause neurodegeneration (reviewed in Bond et al., 2020 [8]). Despite this, it is unclear how C9orf72 hexanucleotide repeats lead to eIF2α phosphorylation and whether this is driven by specific DPR species. To explore this we utilised our previously characterised *Drosophila* models expressing 1000-repeat DPRs to examine eIF2α phosphorylation state at time points previously shown to be pre- and post-motor decline (7- and 21-days post eclosion) [4, 47, 48]. Pan-neuronal (nSyb-Gal4) expression of AP(1000), PR(1000), GR(1000) or GA(1000) had no significant effect on the abundance of total eIF2α in the brains of flies at 7 or 21 days post-eclosion, when compared to controls (Fig. 1a, b, c). While pan-neuronal expression of AP(1000), PR(1000), or GA(1000) also showed no significant difference in the levels of p-eIF2α, compared to controls, pan-neuronal expression of GR(1000) led to a significant increase in the abundance of p-eIF2α in the brains of flies at both 7 (*p* = 0.0003) and 21 days post eclosion (*p* = 0.0045) (Fig. 1a, d, e). Elevated p-eIF2α levels were also reflected in a significant (day 7: *p* = 0.0427 and day 21: *p* = 0.0034) increase in the p-eIF2α:eIF2α ratio observed in the brains of flies pan-neuronally expressing GR(1000), compared to controls, which was not seen with other DPRs (Fig. 1a, f, g ). Statistical comparison between ages reveals no significant difference in total eIF2α, p-eIF2α or p-eIF2α:eIF2α ratio between 7 and 21 days for any genotype (Table S1.). Taken together these results demonstrate pan-neuronal expression of GR(1000) results in a sustained elevation of eIF2α phosphorylation. Having previously shown motor-dysfunction does not occur until later ages in these flies [48], these observations suggest eIF2α phosphorylation is an early event, preceding motor dysfunction.

### Inhibition of the integrated stress response alleviates impaired motor function in *Drosophila* pan-neuronally expressing GR(1000)

Phosphorylation of eIF2α is a common and convergent point of the ISR. It is tightly regulated by specific phosphatases and serine/threonine eIF2α kinases, each of which responds to distinct physiological and pathological stimuli. To explore whether eIF2α phosphorylation merely correlates with, or is an important mediator of, GR(1000)-induced toxicity we utilised the genetic tractability of *Drosophila* to target specific components of the ISR. We then examined the effect on established phenotypes in flies pan-neuronally expressing GR(1000).

**Fig. 2:**
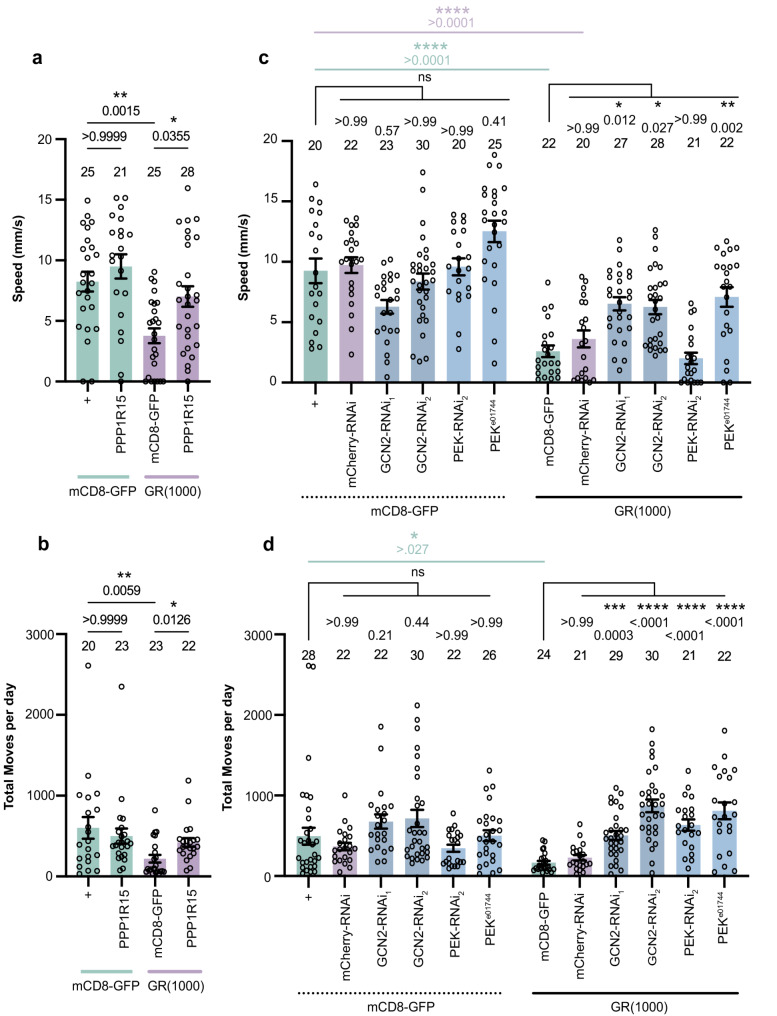
Inhibition of the integrated stress response alleviates motor and activity deficits in *Drosophila* GR(1000) models. **a**–**b** Overexpression of the eIF2α phosphatase PPP1R15 rescues impaired climbing (**a**) and activity (**b**) phenotypes in *Drosophila* pan-neuronally (nSyb-Gal4) expressing GR(1000) at 21 days post-eclosion. Error bars = SEM, Kruskall-Wallis with post-hoc Dunn’s comparison to controls * *p* < 0.05, ** *p* < 0.01. Sample size (n) is reported above each bar. **c**–**d** Knockdown of eIF2α kinases GCN2 and PEK alleviate impaired climbing (**c**) and activity (**d**) phenotypes in *Drosophila* pan-neuronally (nSyb-Gal4) expressing GR(1000) at 21 days post-eclosion. Error bars = SEM, Kruskall-Wallis with post-hoc Dunn’s comparison to controls * *p* < 0.05, ** *p* < 0.01, *** *p* < 0.001, **** *p* < 0.0001. Sample size (n) is reported above each bar

Following the resolution of cellular stress, p-eIF2α is dephosphorylated by the serine/threonine protein phosphatase 1 (PP1). PP1 is a ubiquitous, promiscuous phosphatase and is directed to dephosphorylate p-eIF2α by Protein Phosphatase 1 Regulatory Subunit 15 A (PPP1R15A). Previous studies have demonstrated that overexpression of the *Drosophila PPP1R15A* orthologue, *PPP1R15*, is sufficient to dephosphorylate p-eIF2α and inhibit the ISR [27]. Having confirmed *PPP1R15* overexpression, which increases *PPP1R15* expression by ~ 78% (Fig. S1a), significantly (*p* = 0.006) reduced the elevated p-eIF2α:eIF2α ratio in flies pan-neuronally expressing GR(1000) (Fig. S1d, e) we observed that pan-neuronal co-expression of *PPP1R15* significantly (*p* = 0.0355) alleviated previously characterised climbing impairments in these flies, compared to those co-expressing GR(1000) with a Gal4 titration control (mCD8-GFP) (Fig. 2a). A titration control is used to control for the effect of driving multiple transgenes under the control of a single Gal4 driver. Climbing assays were performed at 21 days post-eclosion; a timepoint at which we previously showed pan-neuronal expression of GR(1000) caused significant motor impairment [48]. Using activity monitors we also showed that at 21 days post-eclosion flies pan-neuronally expressing GR(1000) displayed significantly (*p* = 0.0059) fewer movements, in a 24 h period, than control flies (Fig. 2b), consistent with our previously published data [46]. Critically this reduction in movement was rescued by co-expression of *PPP1R15* (Fig. 2b).

Phosphorylation of mammalian eIF2α is regulated by four specific serine/threonine eIF2α kinases, each responding to distinct cellular cues. These kinases are eukaryotic Translation Initiation Factor 2 Alpha Kinases 1,2,3 and 4 (EIF2AK1, EIF2AK2, EIF2AK3 and EIF2AK4), also known as heme-regulated eIF2α kinase (HRI), Protein Kinase R (PKR), PKR-like endoplasmic reticulum kinase (PERK) and general control nonderepressible 2 (GCN2), respectively. While the core components of the ISR are highly conserved between flies and humans, flies only have two eIF2α kinases; GCN2 and PERK/PEK. Using previously established RNAi lines, we demonstrated that while pan-neuronal knockdown of *GCN2*, (~ 16% knockdown (Fig. S1b)), had no significant effect upon climbing ability (Fig. 2c) or activity (Fig. 2d) in control flies, it significantly rescued climbing (Fig. 2c) and activity impairments (Fig. 2d), as well as the elevated p-eIF2α:eIF2α ratio (Fig. S1d, e), in GR(1000) flies. Pan-neuronal co-expression of a control RNAi (mCherry-RNAi) with GR(1000) had no effect on either climbing or activity phenotypes (Fig. 2c, d). In contrast to *GCN2*, pan-neuronal knockdown of *PERK/PEK* (~ 21% knockdown (Fig. S1c)) ameliorated reduced activity, but not climbing defects in GR(1000) flies (Fig. 2c, d). It also only showed a partial rescue of the elevated p-eIF2α:eIF2α ratio, showing no significant difference to either controls or GR(1000) expressing flies (Fig. S1). Global knockdown of *PERK/PEK* using an established mutant loss-of-function allele (*PEK*^*e01744*^), however, was sufficient to reduce both climbing and activity deficits in GR(1000) flies, suggesting that climbing impairments may be underpinned by non-cell autonomous *PERK/PEK* activation by neuronal GR(1000). *PEK*^*e01744*^ *also* showed a partial rescue of the elevated p-eIF2α:eIF2α ratio, showing no significant difference to the control and a notable, albeit not significant (*p* = 0.0675), decrease compared to GR(1000) expressing flies (Fig. S1d, e). Neither pan-neuronal expression of *PEK*-RNAi nor *PEK*^*e01744*^ had any significant effect on climbing or activity in control flies. Taken together this data suggests that inhibition of the ISR at the level of eIF2α phosphorylation/dephosphorylation can ameliorate toxic phenotypes downstream of neuronal polyGR. Furthermore, the observation that *GCN2* knockdown or *PPP1R15* overexpression showed a more significant effect on elevated p-eIF2α:eIF2α ratio that *PEK* knockdown may suggest that GR(1000) toxicity is driven predominantly through GCN2 dependent activation of the ISR.

### Arginine-rich DPRs co-localise with and enhance the accumulation of the stress/RNP granules

**Fig. 3:**
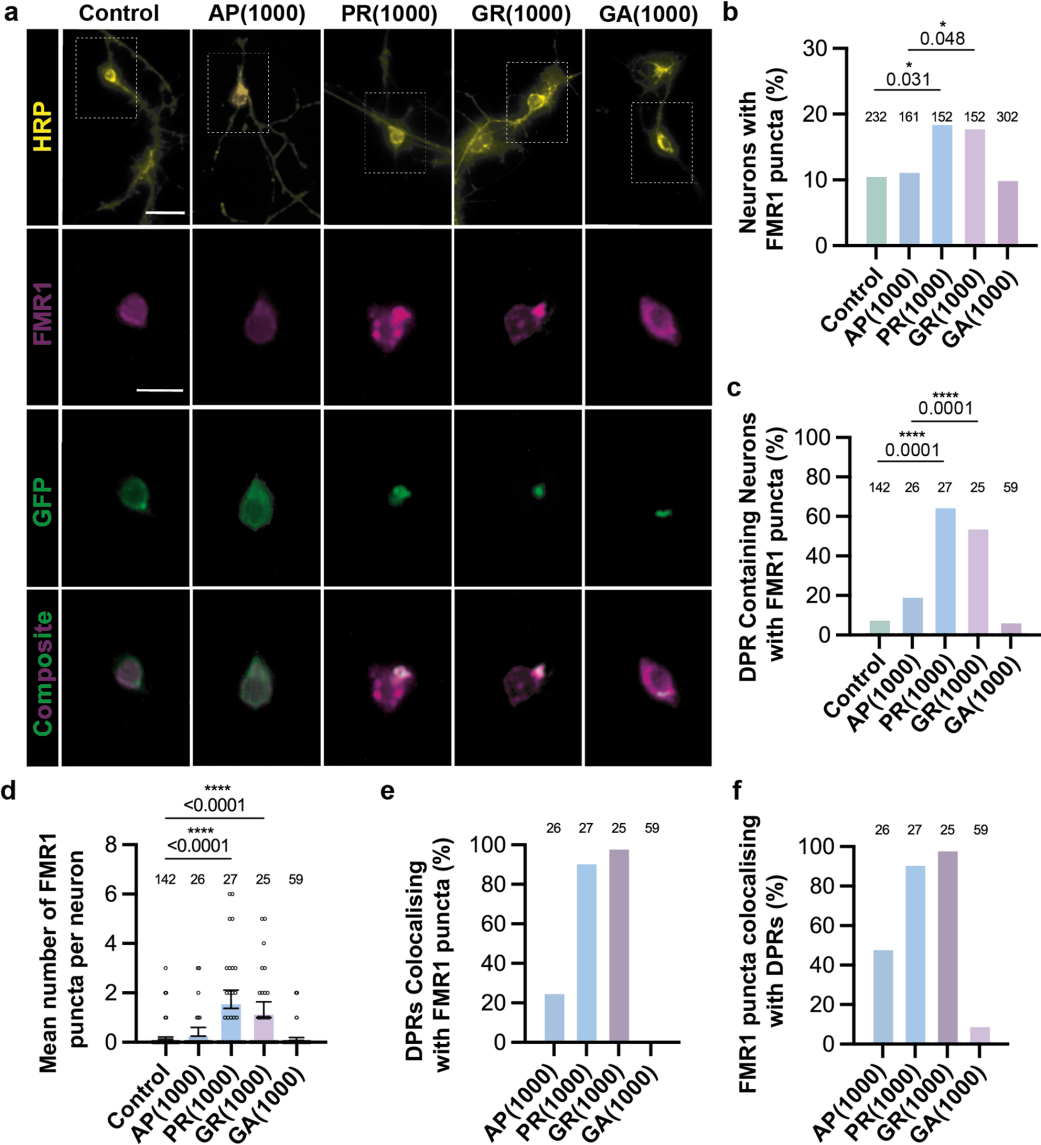
Pan-neuronal expression of arginine rich DPRs leads to accumulation of the stress granule marker FMR1 in *Drosophila* neurons. **a** Representative micrographs of *Drosophila* primary neurons from flies pan-neuronally (nSyb-Gal4) expressing 1000-repeat DPRs, or an mCD8-GFP control. Neurons are labelled with the neuronal marker anti-HRP, anti-GFP (labelling GFP-tagged DPRs) and anti-FMR1. Scale bars = 10 μm. **b** Quantification of the percentage of neurons within primary cultures pan-neuronally (nSyb-Gal4) expressing 1000-repeat DPRs, or an mCD8-GFP control, displaying FMR1 positive puncta. Error bars = SEM, Chi-Squared with Bonferroni correction * *p* < 0.05. Sample size (n) is reported above each bar. **c** Quantification of the percentage of primary neurons displaying DPR aggregates, when pan-neuronally (nSyb-Gal4) expressing 1000-repeat DPRs, or an mCD8-GFP control, containing FMR1 positive puncta. Error bars = SEM, Chi-Squared with Bonferroni correction **** *p* < 0.0001. Sample size (n) is reported above each bar. **d** Quantification of the mean number of FMR1 positive puncta in primary neurons expressing (nSyb-Gal4) 1000-repeat DPRs, or an mCD8-GFP control. Sample size (n) is reported above each bar. **e** The percentage of DPR aggregates colocalising with FMR1 in primary neurons expressing (nSyb-Gal4) 1000-repeat DPRs, or an mCD8-GFP control. Sample size (n) is reported above each bar. **f** The percentage of FMR1 puncta colocalising with DPR aggregates in primary neurons expressing (nSyb-Gal4) 1000-repeat DPRs, or an mCD8-GFP control. Sample size (n) is reported above each bar

**Fig. 4:**
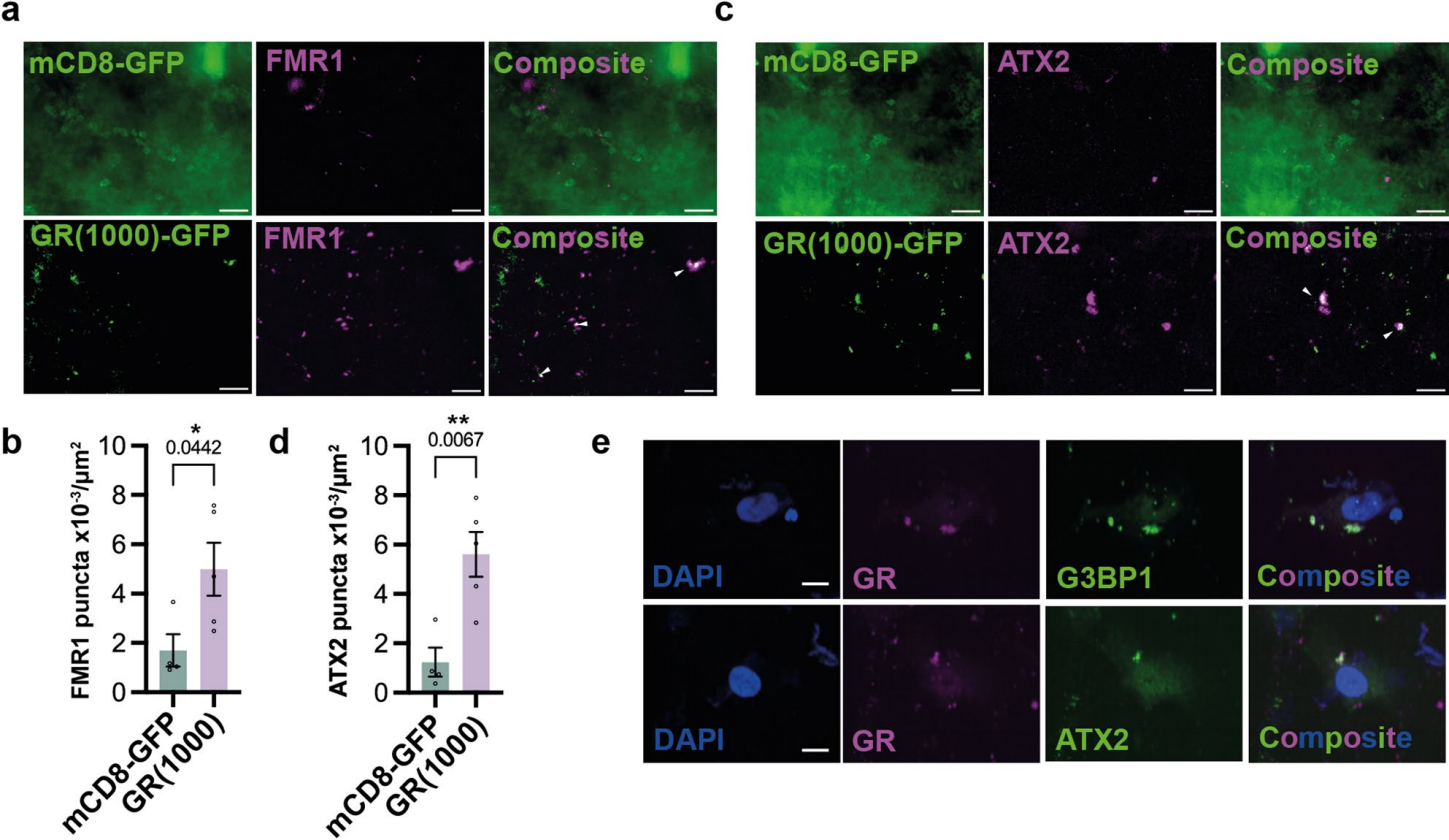
Accumulation of stress granule markers and colocalisation with PolyGR in *Drosophila* brains and patient-derived motor neurons. **a** Representative micrographs showing accumulation of FMR1 and PolyGR in the brains of *Drosophila* pan-neuronally (nSyb-Gal4) expressing GR(1000), or an mCD8-GFP control at 28 days post-eclosion, following heat stress (37 °C for 2 h prior to dissection). Scale bars = 20 μm. **b** Quantification of the number of FMR1 positive puncta in the brains of *Drosophila* pan-neuronally (nSyb-Gal4) expressing GR(1000), or an mCD8-GFP control at 28 days post-eclosion, following heat stress (37 °C for 2 h prior to dissection). Error bars = SEM, unpaired T-test * *p* < 0.05. **c** Representative micrographs showing accumulation of ATX2 and PolyGR in the brains of *Drosophila* pan-neuronally (nSyb-Gal4) expressing GR(1000), or an mCD8-GFP control at 28 days post-eclosion, following heat stress (37 °C for 2 h prior to dissection). Scale bars = 20 μm. **d** Quantification of the number of ATX2 positive puncta in the brains of *Drosophila* pan-neuronally (nSyb-Gal4) expressing GR(1000), or an mCD8-GFP control at 28 days post-eclosion, following heat stress (37 °C for 2 h prior to dissection). Error bars = SEM, unpaired T-test ** *p* < 0.01. **e** Representative micrographs showing colocalisation of PolyGR with stress granule markers G3BP1 and ATX2 in day 40 C9orf72 repeat expansion patient-derived motor neurons. Scale bars = 10 μm

Phosphorylation of eIF2α initiates the formation of stress granules in response to a range of cellular stresses. While stress granules play a protective role, accumulating evidence suggests that chronic stress granule accumulation may contribute towards neurodegeneration in ALS and FTD. Having observed increased eIF2α phosphorylation and shown that inhibition of the ISR can ameliorate degenerative phenotypes in flies pan-neuronally expressing GR(1000), we asked whether pan-neuronal expression of specific DPR species led to the accumulation of stress granules within neurons. Using the cellular resolution of primary neurons, we showed neuronal cultures from flies pan-neuronally expressing either PR(1000) or GR(1000) demonstrated a significant increase (*p* = 0.031 and *p* = 0.048, respectively) in the percentage of neurons containing puncta positive for the established stress granule marker FMR1, compared to controls (Fig. 3a, b). Anti-HRP was used as an established neuronal marker in *Drosophila* [43, 45], while anti-GFP was used to label GFP-tagged DPRs. Cultures from flies pan-neuronally expressing GA(1000) or AP(1000) showed no significant difference to controls. The percentage of neurons showing FMR1 positive stress granules was even greater when looking specifically at those with visible DPR accumulations (Fig. 3c). In addition to seeing a greater number of neurons with FMR1 positive puncta when PR(1000) or GR(1000) were pan-neuronally expressed, these neurons also showed a significant (*p* < 0.0001) increase in the number of FMR1 positive puncta observed within each neuron (Fig. 3d). The majority of these FMR1 positive puncta were observed to colocalise with PR or GR, and vice versa (Fig. 3e, f). The alternative stress granule marker eukaryotic translation initiation factor 4E (eIF4E) was also observed to accumulate in primary neurons when GR(1000) was pan-neuronally expressed, or when control cells were treated with chemical stressor sodium arsenite (NaAsO_2_) (Fig. S2a, b, c). A significant increase in the number of FMR1 positive stress granules was also observed in vivo, in the brains of 28 day old flies pan-neuronally expressing GR(1000), but not other DPRs, compared to controls (Fig. S2d, e). To explore this further, we examined whether heat stress induced stress granule accumulation, as observed in previous studies and control flies (Fig. S2f, g), could potentiate stress granule formation in flies pan-neuronally expressing GR(1000). Under non-stressed conditions flies pan-neuronally expressing GR(1000) displayed ~ 112% more FMR1 positive stress granules than GFP expressing controls (Fig. S2d, e). However, following heat stress both GFP controls and GR(1000) expressing flies showed an increase in the number of FMR1 positive puncta (Fig. 4a, b), with GR(1000) flies displaying ~ 194% more FMR1 positive stress granules than GFP expressing controls. Similarly, following heat stress, flies pan-neuronally expressing GR(1000) displayed ~ 354% more puncta positive for the stress granule marker ATX2 than GFP expressing controls (Fig. 4c, d). Taken together these results suggest flies pan-neuronally expressing GR(1000) show a greater sensitivity to stress, displaying increased stress granule accumulation under basal and stress conditions, than controls. Additionally, a number of FMR1- and ATX2-positive puncta were observed to co-localize with GR(1000) (Fig. 4a, c). To explore whether this phenotype was conserved in human neurons, we examined C9orf72 repeat expansion patient-derived iPSC motor neurons. Consistent with our findings in *Drosophila*, we observed strong co-localization between ATXN2 and PolyGR in these neurons (Fig. 4e). ATXN2 and PolyGR showed a positive correlation in signal intensity (thresholded Pearson’s *R* = 0.63), while thresholded Manders’ overlap coefficients demonstrated that 93.9% of the PolyGR signal overlapped with ATXN2 (tM2 = 0.939) and 49.1% of the ATXN2 signal overlapped with PolyGR (tM1 = 0.491). PolyGR was also observed to co-localize with the stress granule marker G3BP1 (Fig. 4e), showing a strong positive correlation in signal intensity (thresholded Pearson’s *R* = 0.85). Furthermore, thresholded Manders’ overlap coefficients demonstrated a high degree of co-occurrence between the two proteins, with 94.2% of the PolyGR signal overlapping with G3BP1 (tM2 = 0.942) and 82.8% of the G3BP1 signal overlapping with PolyGR (tM1 = 0.828). Approximately 20% of neurons in culture presented with visible GR inclusions. These results align with previous proteomic studies demonstrating interactions between PolyGR and several stress/RNP granule proteins, including G3BP1 and ATXN2 [25]. Furthermore, they confirm the conservation of these pathological phenotypes between *Drosophila* models and human neurons.

### Inhibition of the integrated stress response reduces the number of FMR1 positive puncta observed in *Drosophila* pan-neuronally expressing GR(1000)

**Fig. 5:**
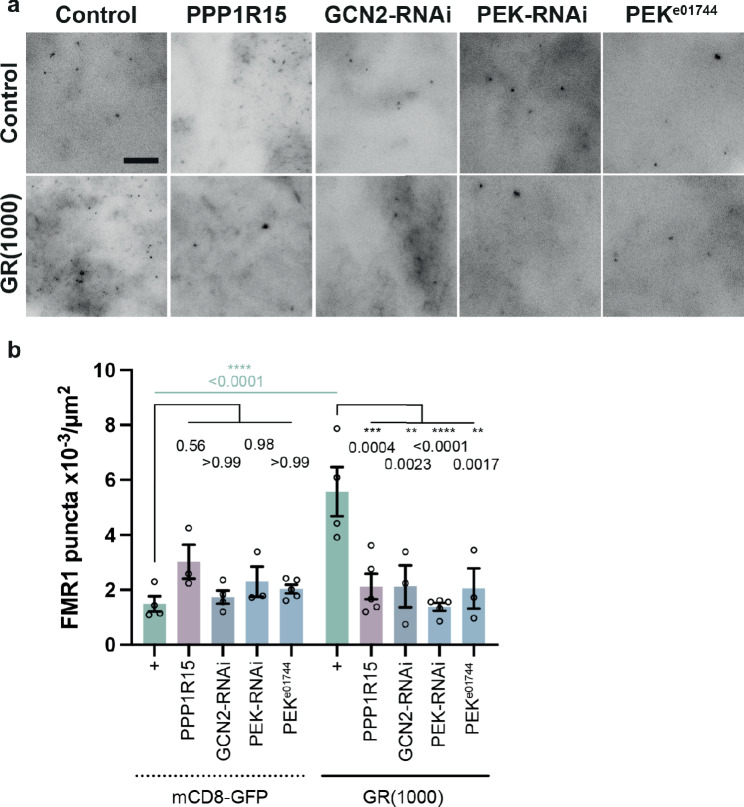
Inhibition of the integrated stress response reduces the number of FMR1 positive puncta observed in *Drosophila* pan-neuronally expressing GR(1000). **a** Representative micrographs showing accumulation of FMR1 in the brains of *Drosophila* pan-neuronally (nSyb-Gal4) expressing GR(1000), or an mCD8-GFP control at 21 days post-eclosion. Scale bars = 25 μm. **b** Quantification of the number of FMR1 positive puncta in the brains of *Drosophila* pan-neuronally (nSyb-Gal4) expressing GR(1000), or an mCD8-GFP control at 21 days post-eclosion. Error bars = mean ± SEM. ANOVA with post-hoc Šídák’s multiple comparison test, ** *p* < 0.01, *** *p* < 0.001, **** *p* < 0.0001

Having shown that Inhibition of the ISR alleviates impaired motor function in *Drosophila* pan-neuronally expressing GR(1000), we asked whether ISR inhibition could also reduce the stress/RNP granule accumulation observed in these flies. Overexpression of PPP1R15, knockdown of GCN2 and knockdown of PEK all significantly reduced the number of FMR1 positive stress/RNP granules observed in the brains of *Drosophila* pan-neuronally expressing GR(1000) at 21 days post-eclosion (Fig. 5).

### Knockdown of ATX2 rescues impaired motor function, seizure phenotypes and stress granule accumulation in *Drosophila* pan-neuronally expressing GR(1000)

**Fig. 6:**
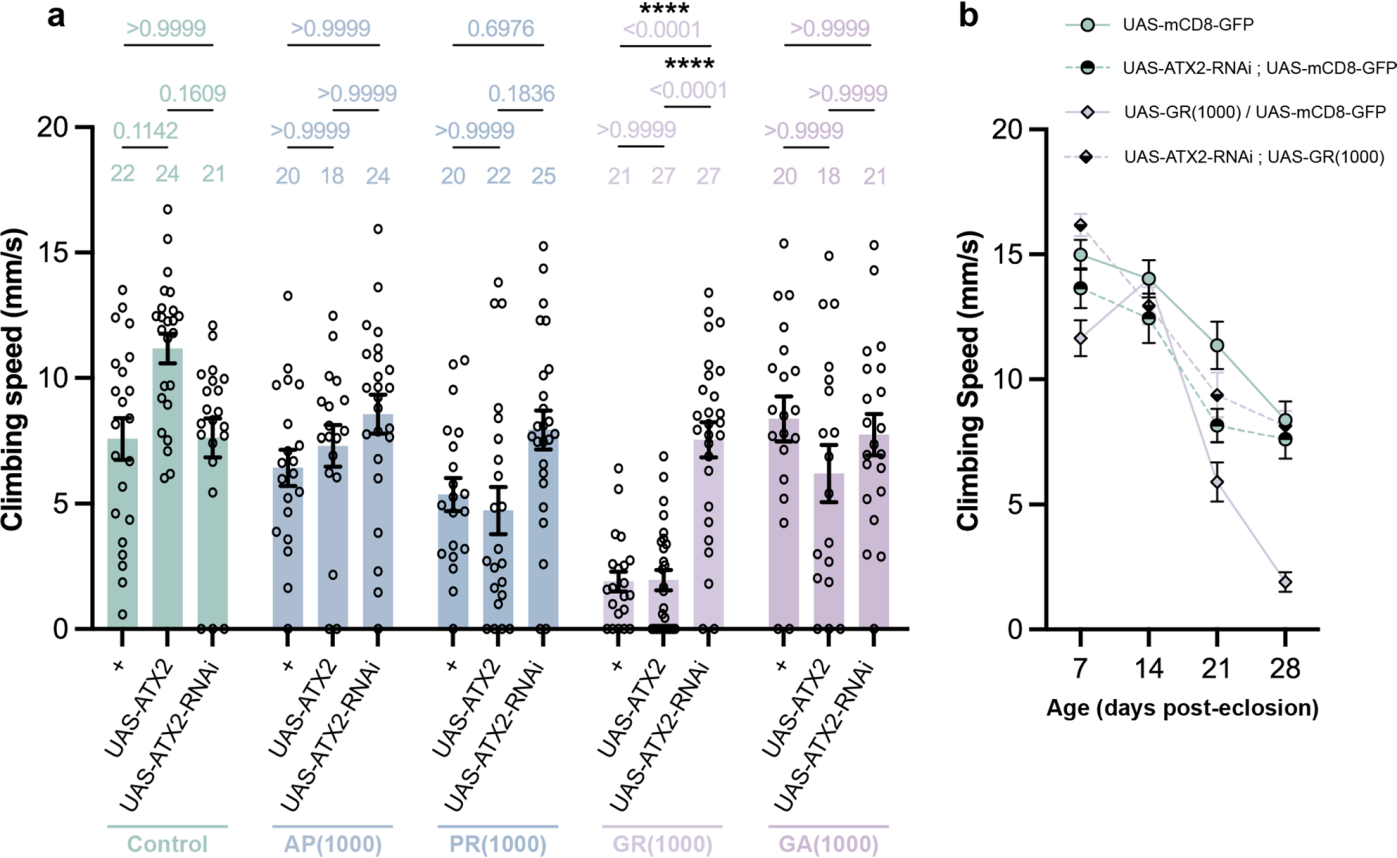
Knockdown of ATX2 alleviates climbing deficits in *Drosophila* GR(1000) models. **a** Quantification of climbing speed in flies pan-neuronally (nSyb-Gal4) co-expressing 1000-repeat DPRs, or an mCD8-GFP control (Control), with either a Gal4 titration control ( + = mCD8-GFP), UAS-ATX2 or UAS-ATX2-RNAi, at 28 Days post-eclosion. Error bars = SEM, ANOVA with post-hoc Tukey test, **** *p* < 0.0001. Sample size (n) is reported above each bar. **b** Quantification of climbing speed in flies pan-neuronally (nSyb-Gal4) co-expressing GR(1000), or an mCD8-GFP control, with either a Gal4 titration control (mCD8-GFP) or UAS-ATX2-RNAi, at 7, 14, 21 and 28 Days post-eclosion. Error bars = SEM

Previous studies have shown that targeted reduction in RNP-/stress-granule formation can convey neuroprotection. *ATXN2*, an established ALS risk gene [14], is essential for normal RNP- and stress-granule formation, mediating their assembly though intrinsically disordered regions (IDRs). Indeed deletion of IDRs in ATX2 has been shown to reduce toxicity observed when FUS or 50-repeat polyGR are expressed in the *Drosophila* eye [2]. In addition, knockdown of *ATXN2* has previously been shown to be protective in yeast, fly and mouse TDP-43 models of FTD/ALS [3, 14]. Having observed the accumulation of RNP-/stress-granules in GR(1000) expressing flies, we asked whether knockdown of *ATX2* was sufficient to alleviate established phenotypes in these models. Pan-neuronal RNAi-mediated, knockdown of *ATX2* had no significant effect on climbing ability in control, AP(1000), PR(1000) or GA(1000) flies at 28 days post-eclosion (Fig. 6a). However, *ATX2* knockdown significantly (*p* < 0.0001) rescued impaired climbing ability in flies pan-neuronally expressing GR(1000) (Fig. 6a, b). A rescue of impaired climbing ability in flies pan-neuronally expressing GR(1000) was also observed by knockdown of *ATX2* with a second independent RNAi line (Fig. S3a). Characterisation of knockdown efficiency reveals an ~ 50% reduction in ATX2 protein levels with both RNAi lines (Fig. S3b, c). Pan-neuronal co-expression of a control RNAi line (mCherry-RNAi) with GR(1000) showed no rescue effect (Fig. S3a). In contrast, over-expression of *ATX2* did not potentiate GR(1000) toxicity and had no significant effect on climbing ability in controls or any DPR expressing line (Fig. 6a, b). Similarly, a modifier screen revealed co-expression of human *ATXN2* carrying either non-pathogenic (22 repeat) or pathogenic (32 repeat) polyglutamine expansions had no effect on the toxicity of any DPR species, when expressed in the *Drosophila* eye (Fig. S3d).

**Fig. 7:**
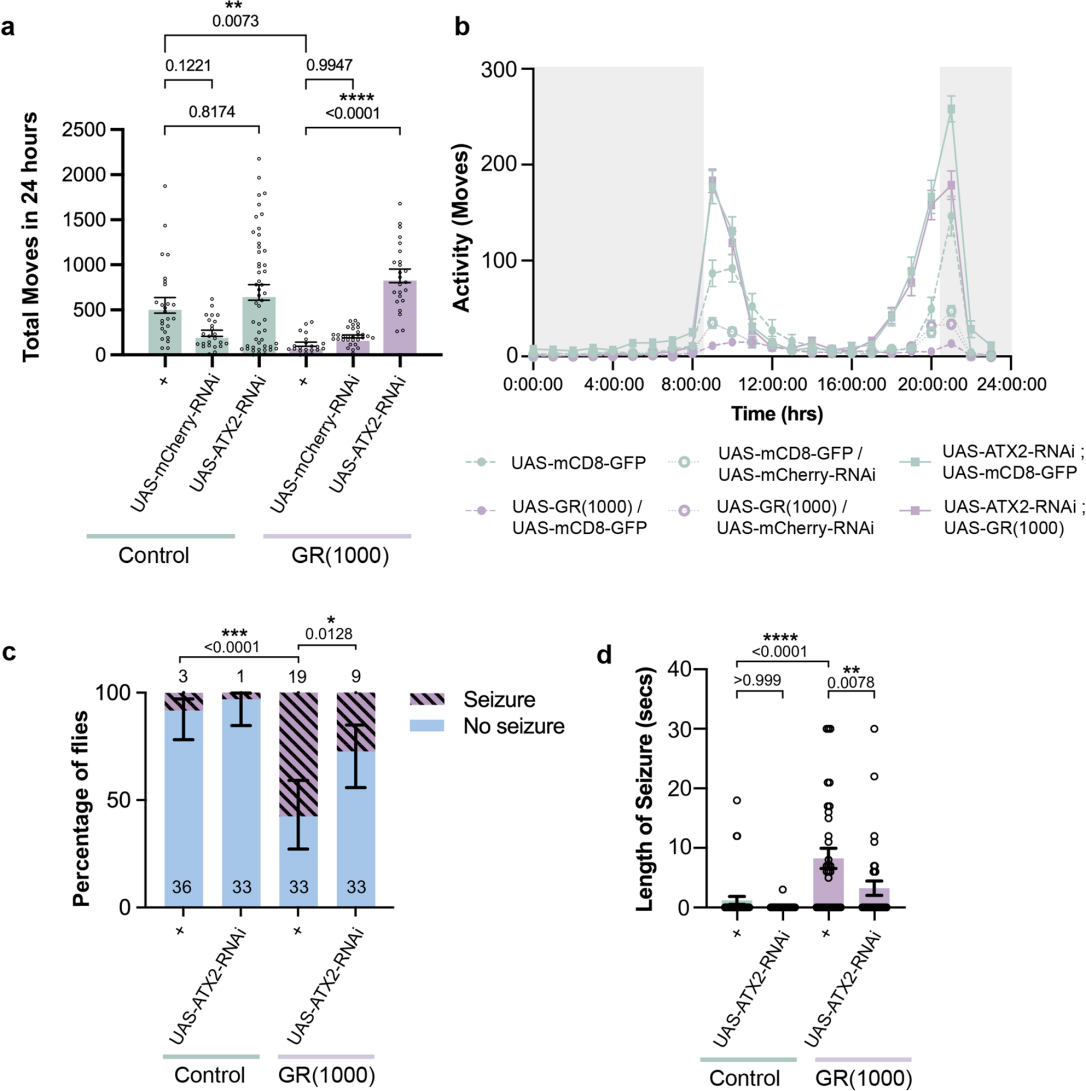
Knockdown of ATX2 alleviates activity and seizure phenotypes in *Drosophila* GR(1000) models. **a** Quantification of activity (Moves made in 24 h) in flies pan-neuronally (nSyb-Gal4) co-expressing GR(1000), or an mCD8-GFP control (Control), with either a Gal4 titration control ( + = mCD8-GFP), an RNAi control (UAS-mCherry-RNAi) or UAS-ATX2-RNAi^1^, at 28 Days post-eclosion. Error bars = SEM, Kruskall-Wallis with post-hoc Dunn’s comparison to controls ** *p* < 0.01, **** *p* < 0.0001. **b** 24 h actogram showing hourly activity (Moves) of flies pan-neuronally (nSyb-Gal4) co-expressing GR(1000), or an mCD8-GFP control (Control), with either a Gal4 titration control ( + = mCD8-GFP), an RNAi control (UAS-mCherry-RNAi) or UAS-ATX2-RNAi^1^, at 28 Days post-eclosion. **c** Quantification of the percentage of flies displaying seizure phenotypes following mechanical (vortex) stimulation in flies pan-neuronally (nSyb-Gal4) co-expressing GR(1000), or an mCD8-GFP control (Control), with either a Gal4 titration control ( + = mCD8-GFP) or UAS-ATX2-RNAi^1^ at 28 Days post-eclosion. Error bars = SEM, Chi-Squared with Bonferroni correction * *p* < 0.05, *** *p* < 0.001. Number of flies seizing is reported above each bar and flies not seizing within the bar. **d** Quantification of the length of seizures observed following mechanical (vortex) stimulation in flies pan-neuronally (nSyb-Gal4) co-expressing GR(1000), or an mCD8-GFP control (Control), with either a Gal4 titration control ( + = mCD8-GFP) or UAS-ATX2-RNAi^1^ at 28 Days post-eclosion. Error bars = SEM, Kruskall-Wallis with post-hoc Dunn’s comparison to controls ** *p* < 0.01, **** *p* < 0.0001

Having shown pan-neuronal knockdown of *ATX2* is sufficient to alleviate impaired climbing ability in flies pan-neuronally expressing GR(1000) we next asked whether *ATX2* knockdown could also rescue the reduced activity observed in these flies. Pan-neuronal expression of GR(1000) with a Gal4 titration control (mCD8-GFP) resulted in a significant (*p* = 0.0073) reduction in the activity of flies observed over a 24 h period, compared to controls (Fig. 7a, b). Pan-neuronal knockdown of *ATX2* in a GR(1000) background led to a significant (*p* < 0.0001) rescue of reduced activity. In contrast, pan-neuronal knockdown of *ATX2* had no significant effect on the activity of control flies. A control RNAi (mCherry RNAi) had no significant effect on the activity of control or GR(1000) flies.

Previously we found that pan-neuronal co-expression of specific combinations of DPR species induced age-dependent “bang-sensitive” seizure phenotypes. We have subsequently found that subjecting flies to more rigorous mechanical stimulation, by employing an established vortex stimulation assay [29], leads to a significant number of flies pan-neuronally expressing GR(1000) presenting with seizures, at 14 days post-eclosion (Fig. S3e). Pan-neuronal expression of other DPR species did not result in a significant number of seizures being observed (Fig. S3e). We therefore asked whether knockdown of *ATX2* could alleviate seizure phenotypes in flies pan-neuronally expressing GR(1000). At 28 days post-eclosion a significant percentage (~ 58%, *p* < 0.0001) of flies pan-neuronally expressing GR(1000), with a Gal4 titration control, presented with seizures (Fig. 7c). Pan-neuronal co-expression of *ATX2-*RNAi with GR(1000) resulted in a significant reduction (~ 27%, *p* = 0.0128) in the number of flies exhibiting seizures and significantly ( *p* = 0.0078) decreased seizure duration (Fig. 7d).

**Fig. 8:**
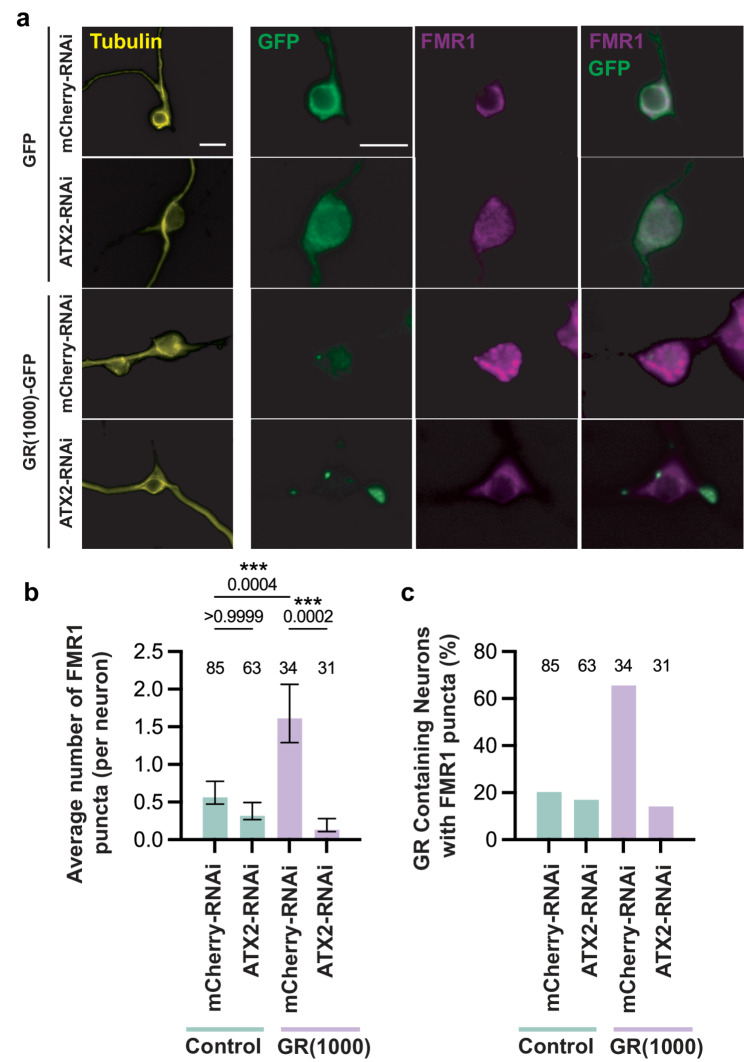
Knockdown of ATX2 reduces the number of FMR1 positive stress granules observed in GR(1000) expressing primary neurons. **a** Representative micrographs of *Drosophila* primary neurons from flies pan-neuronally (nSyb-Gal4) co-expressing GR(1000), or an mCD8-GFP control, with either an RNAi control (UAS-mCherry-RNAi) or UAS-ATX2-RNAi^1^. Scale bars = 5 μm. **b** Quantification of the mean number of FMR1 positive puncta in primary neurons from flies pan-neuronally (nSyb-Gal4) co-expressing GR(1000), or an mCD8-GFP control, with either an RNAi control (UAS-mCherry-RNAi) or UAS-ATX2-RNAi^1^. Error bars = SEM, Kruskall-Wallis with post-hoc Dunn’s comparison to controls *** *p* < 0.001. Sample size (n) is reported above each bar. **c** Quantification of the percentage of neurons pan-neuronally (nSyb-Gal4) co-expressing GR(1000), or an mCD8-GFP control, with either an RNAi control (UAS-mCherry-RNAi) or UAS-ATX2-RNAi^1^ containing FMR1 positive puncta. Error bars = SEM, Chi-Squared with Bonferroni correction **** *p* < 0.0001. Sample size (n) is reported above each bar

Having shown that knockdown of *ATX2* was sufficient to ameliorate climbing deficits, reduced activity and seizure phenotypes in *Drosophila* pan-neuronally expressing GR(1000), we looked to determine whether *ATX2* knockdown could also reduce the stress/RNP granule accumulation phenotype observed in GR(1000) flies, or whether the rescue of motor and seizure phenotypes was independent of this. Knockdown of *ATX2* in controls had no effect on the mean number of FMR1-positive puncta per neuron or the number of neurons containing FMR1-positive stress/RNP granules per GFP positive neuron (Fig. 8a, b, c). In contrast, *ATX2* knockdown in GR(1000) expressing neurons led to a significant reduction in both the number of FMR1-positive puncta per neuron (Fig. 8a, b, *p* = 0.0002) and the percentage of neurons with GR inclusions containing FMR1-positive stress/RNP granules (Fig. 8a–c). *ATX2* knockdown also significantly (*p* = 0.0025) reduced the increased p-eIF2α/eIF2α ratio observed in flies pan-neuronally expressing GR(1000) (Fig. S1d, e), suggesting that despite ATX2 dependent stress granule formation being downstream of eIF2α phosphorylation, and the ISR, persistent accumulation of stress granules may potentiate ISR activation.

### Knockdown of ATXN2 ameliorates perturbations to neuronal morphology in mouse primary neurons transduced with PolyGR

**Fig. 9:**
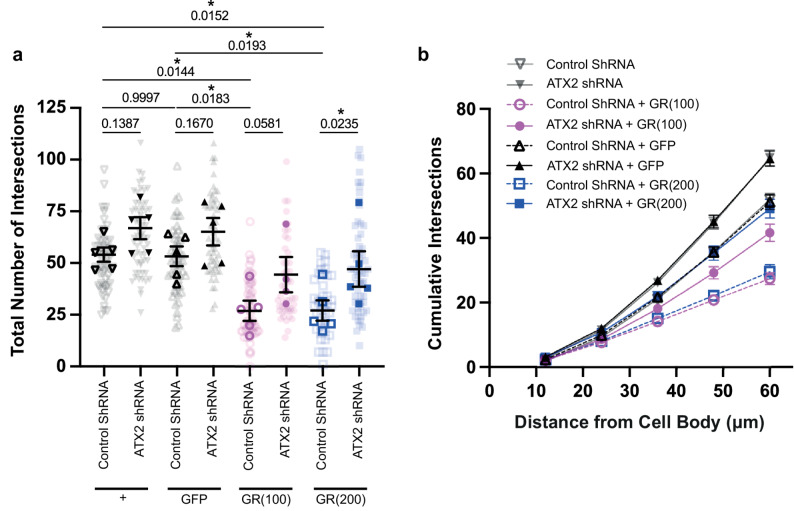
Knockdown of ATX2 ameliorates neuronal morphology defects in mouse primary neurons transduced with PolyGR. **a** Quantification of the dendritic complexity (total number of intersections) of mouse primary neurons co-transduced with either GFP, GR100 or GR200 and either a control scrambled shRNA or ATXN2-shRNA. Shaded/transparent shapes represent individual cells with solid/opaque shapes showing the mean of each biological replicate. Statistics were performed on the means of biological replicates, 2-way ANOVA with post-hoc Tukey test, * *p* < 0.05. **b** Quantification of the dendritic complexity (cumulative number of intersections) of mouse primary neurons co-transduced with either GFP, GR100 or GR200 and either a control scrambled shRNA or ATXN2-shRNA

Alterations to neuronal morphology, including the reduced complexity of dendritic arborisation has been observed in C9orf72 and DPR models [17, 22]. In our preliminary studies we observed an apparent reduction in dendritic complexity in mouse primary neurons transduced with either 100 or 200 repeats of PolyGR. We therefore looked to determine whether knockdown of *ATXN2* was sufficient to alleviate reduced dendritic complexity in these models. Transduction of GR(100) with a control shRNA caused a significant reduction in dendritic complexity when compared to cells transduced with a control shRNA alone (*p* = 0.0144) or co-transduced with GFP and the control shRNA (*p* = 0.0183) (Fig. 9a, b). Reduced complexity was also observed when neurons were co-transduced with GR(200) and the control shRNA (*p* = 0.0152 vs. control shRNA and *p* = 0.0193 vs. GFP + control shRNA) (Fig. 9a, b). Co-transduction of GR(100) with an ATXN2 shRNA resulted in a partial, albeit non-significant (*p* = 0.0581), rescue of this phenotype, whilst co-transduction of GR(200) with an ATXN2 shRNA resulted in a significant (*p* = 0.0235) partial rescue (Fig. 9a, b). These findings highlight a conserved role for ATXN2 knockdown in conveying neuroprotection in both *Drosophila* and mammalian models.

## Discussion

In this study we demonstrate that neuronal GR(1000) leads to a significant increase in the phosphorylation of eIF2α, the core of the ISR, as well as accumulation of stress granule markers, FMR1 and ATX2, in the brains of *Drosophila*. In addition, we find stress granule markers colocalise with GR aggregates in *Drosophila* brains, primary neurons and patient-derived motor neurons. These findings suggest that activation of the ISR and accumulation of stress granules, two intrinsically linked pathways previously implicated in C9orf72-related FTD/ALS, may be driven by polyGR. Critically, we demonstrate that genetic inhibition of the ISR or ATXN2, a key regulator of stress granules, can alleviate previously established phenotypes, including impaired motor function and activity deficits, in *Drosophila* pan-neuronally expressing GR(1000). Supporting this we show knockdown of ATXN2 to be protective in mouse primary neurons transduced with GR(200). Taken together these observations demonstrate the role of stress granules and the wider ISR in the pathophysiology of C9orf72-related FTD/ALS and reveal ATXN2 and the ISR to be promising targets for potential treatment of C9orf72-related FTD/ALS, and perhaps FTD/ALS spectrum disorders more broadly.

Converging lines of evidence indicate that both the ISR and dysregulated stress granule dynamics contribute to the pathogenesis of ALS and FTD [10, 15, 18, 20, 32, 39, 49, 50]. These pathways are intrinsically linked with phosphorylation of the central ISR component eIF2α, triggering stress granule formation [32]. Elevated levels of p-eIF2α and stress granule accumulation have previously been demonstrated in both C9orf72 patient-derived cell models and patient tissue, however the mechanisms leading to this and whether it is underpinned by specific DPR species remains unclear [18, 20, 32, 39, 49, 50]. eIF2α phosphorylation and ISR activation have been shown to enhance RAN translation and DPR production, whilst the G_4_C_2_ repeat expansion, in turn, promotes ISR activation, creating a potential toxic feed forward mechanism [10, 15, 32]. Indeed, whilst both the ISR and stress granules are essential for normal cellular function, maintaining cellular homeostasis in response to various cellular stressors, there is increasing evidence to show that chronic activation of the ISR and/or aberrant, prolonged persistence of stress granules can lead to neurotoxicity and neurodegeneration. Accumulation of stress granules may occur either due to a failure to clear stress granules or from chronic ISR activation promoting stress granule formation. In this study the observation that levels of p-eIF2α are elevated in the brains of flies expressing GR(1000), but not those expressing other DPRs, prior to the onset of motor phenotypes suggests ISR activation by polyGR may be an early pathogenic event leading to chronic activation of the ISR, aberrant accumulation of stress granules and downstream toxicity. Whilst, in contrast to GR(1000), we did not observe elevated p-eIF2α in the brains of flies pan-neuronally expressing PR(1000), a previous study from Kramer et al., (2018) showed transcriptional upregulation of genes involved in endoplasmic reticulum stress and the ISR in both mouse primary neurons transduced with PR(50), and K562 cells, a human immortalized lymphoblast cell line, treated with synthetic PR(20) [24]. They also found pre-treatment of PR(20) treated K562 cells with the ISR inhibitor ISRIB improved viability of these cells [24]. This disparity in which DPRs trigger the ISR and downstream toxicity may be attributed to differences in the capacity of in vitro and in vivo systems to mediate cellular stress responses and/or the levels of exogenous stress these systems are exposed to in an experimental setting. Supporting this we show that despite p-eIF2α levels not being elevated in PR(1000) fly brains, we did observe accumulation of stress granules in primary neurons cultured from these flies.

Critically, supporting the ISR as a potential therapeutic target, our genetic dissection suggests the ISR can be targeted at various points, including knockdown of eIF2α kinases *GCN2* or *PEK*, or upregulation of the eIF2α phosphatase *PPP1R15*, to ameliorate motor dysfunction in GR(1000) expressing flies. Taken together these observations support the hypothesis that chronic activation of the ISR leads to neurodegeneration, with ISR inhibition offering an attractive therapeutic target. This is supported by similar observations in other pre-clinical FTD/ALS models. For example, previous studies have shown that the PERK inhibitor GSK2606414 was sufficient to mitigate TDP-43 toxicity in both *Drosophila* and rat primary cortical neurons [23]. Similarly, studies have demonstrated that inhibition of either GCN2 or PERK, or inhibition of the ISR using ISRIB can convey neuroprotection in mouse models of Alzheimer’s disease [26, 31], whilst GSK2606414 was protective in mouse models of Parkinson’s disease [28]. ISR inhibitors such as ISRIB have also been shown to block eIF2α phosphorylation-induced stress granule formation [42]. Collectively these findings highlight the potential for small molecule ISR inhibitors such as ISRIB for the treatment of FTD/ALS spectrum disorders.

In addition to showing that ISR inhibition prevented toxicity in GR(1000) flies we found that knockdown of the stress granule regulator ATX2/ATXN2 was sufficient to mitigate toxicity in both GR(1000) flies and mouse primary neurons transduced with PolyGR. ATXN2 is an RNA binding protein that plays a fundamental role in mRNA translation, RNA regulation, the assembly of processing bodies (P-bodies) and the formation of stress granules. Polyglutamine expansions with the *ATXN2* gene have been implicated in several neurodegenerative diseases, including spinocerebellar ataxia type 2 (SCA2) [19, 38], ALS and FTD [14, 36]. In healthy individuals the *ATXN2* polyglutamine tract typically comprises 22–23 glutamine residues (polyQs). In SCA2 this is expanded to > 35 repeats, with intermediate expansions (24–34 repeats) conferring increased ALS risk. Intermediate expansions have also been detected with higher frequency in bvFTD cases [36]. Previous studies have suggested that intermediate expansions may reduce the clearance of ATXN2, leading to elevated levels and downstream toxicity [14]. However, in this study pan-neuronal overexpression of ATX2 had no effect on established phenotypes observed in our DPR fly models. In addition, expression of human ATXN2 with normal (22Q) and intermediate (32Q) polyglutamine expansions did not modify DPR toxicity, when expressed in the *Drosophila* eye. Flies pan-neuronally expressing GR(1000) also did not show elevated levels of ATX2, suggesting toxicity in these flies is not driven by increased ATX2. In contrast, knockdown of *ATX2* was sufficient to alleviate all phenotypes characterised in the GR(1000) fly model, as well as mouse primary neurons transduced with PolyGR, highlighting *ATX2* knockdown as a potential therapeutic target in ALS/FTD. Indeed, previous studies have shown *ATX2/ATXN2* knockdown to be protective in several TDP-43 models of ALS, including *Drosophila*, yeast, and mice [3, 14]. For example, Becker et al., (2017) demonstrated that knockdown of *ATXN2* in TDP-43 transgenic mice, using small interfering RNAs (siRNAs) or antisense-oligonucleotides (ASO), reduced TDP-43 toxicity by decreasing its transit to, and propensity to aggregate within, stress granules [3]. This resulted in a dose dependent slowing of disease progression, improved motor function and increased survival. This study was key in establishing ATXN2 as a therapeutic target for ALS, and implicating stress granules in the pathophysiology of the disease. The results of preclinical studies targeting *ATXN2* in TDP-43 models has underpinned *ATXN2* as an attractive target for potential therapeutic intervention. Now we show this may also be the case for C9orf72 related FTD/ALS. While a recent clinical trial (NCT04494256) assessing the effectiveness of ATXN2 ASOs for ALS proved unsuccessful, the effectiveness of this approach in pre-clinical models suggests targeting *ATXN2* much earlier may be critical for effective intervention. Indeed, the findings of our study demonstrate that activation of the ISR occurs as an early event, preceding motor and behavioural dysfunction, in *Drosophila* pan-neuronally expressing GR(1000). In addition, while ATXN2 ASOs were shown to effectively reduce ATXN2 levels in cerebral spinal fluid, their ability to reach and sustain therapeutic levels in affected motor neurons may have been insufficient. Further studies will be critical to assess whether earlier intervention, improved delivery, or complementary strategies such as targeting both the ISR and ATXN2 simultaneously, could enhance effectiveness in a clinical setting.

Utilising the genetic tractability of *Drosophila* models, expressing DPRs of a length comparable to the longest repeats observed in C9orf72 patients, this study demonstrates that neuronal PolyGR activates the ISR and promotes stress granule accumulation. These two intrinsically linked pathways have both been implicated in the pathogenesis of ALS and FTD. Thus, our findings demonstrating genetic inhibition of the ISR or ATXN2 conveys neuroprotection underscores these pathways as important therapeutic targets for C9orf72-related FTD/ALS, and possibly other FTD/ALS spectrum disorders.

## Supplementary Information

Below is the link to the electronic supplementary material.

Supplementary Material 1.

Supplementary Material 2.

## Data Availability

The datasets used and/or analysed during the current study are available from the corresponding author on reasonable request.
