# Supplementary material for: Targeting the integrated stress response or Ataxin-2 alleviates neurodegeneration in PolyGR models of C9orf72 associated frontotemporal dementia and amyotrophic lateral sclerosis
Source: Acta Neuropathol Commun. 2026 May 5;14:124. doi: 10.1186/s40478-026-02301-2 (PMC13251143; doi:10.1186/s40478-026-02301-2)

Full Blots

Figure 1:

7 Days Post-eclosion

Lanes are: ladder, control, AP(1000), PR(1000), GR(1000), GA(1000)

Anti-eIF2α

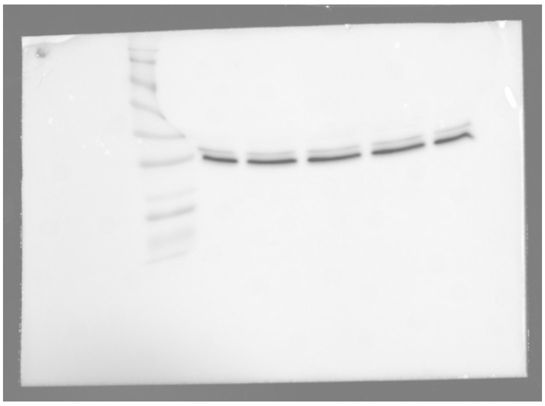

Anti-eIF2α + anti-tubulin

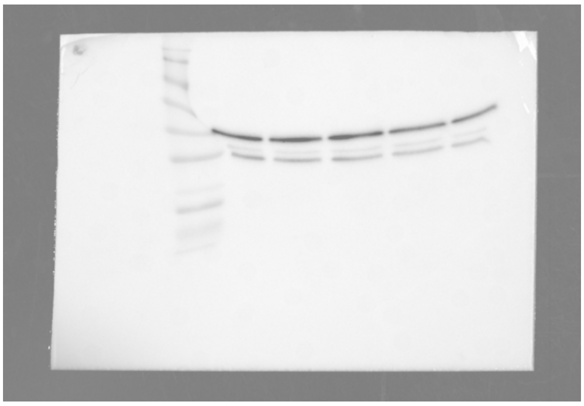

Anti-phospho-eIF2α

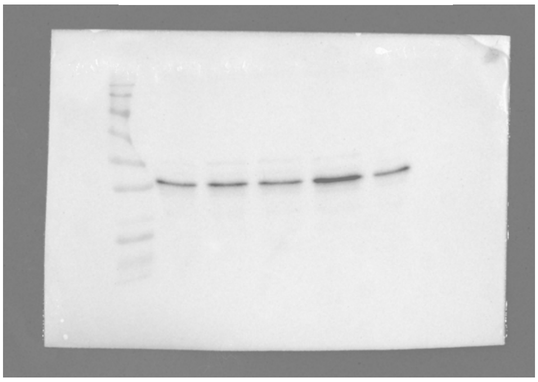

Anti-tubulin

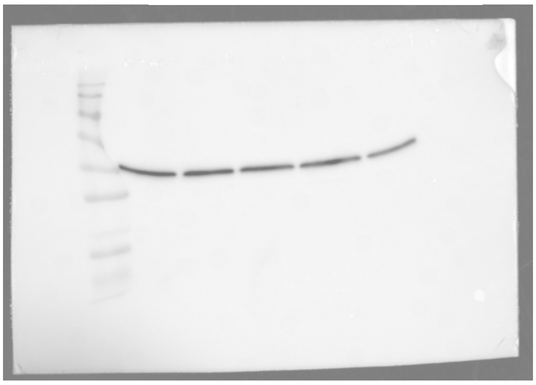

21 Days Post-eclosion

Lanes are: ladder, control, AP(1000), PR(1000), GR(1000), GA(1000)

Anti-eIF2α

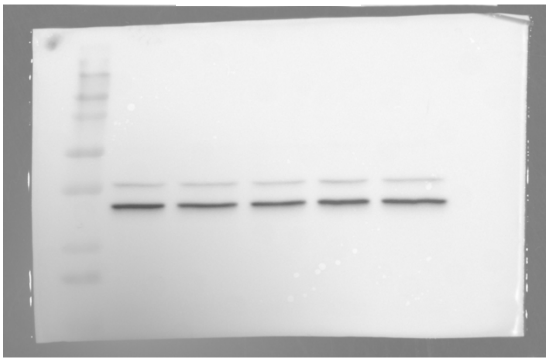

Anti-eIF2α + anti-tubulin

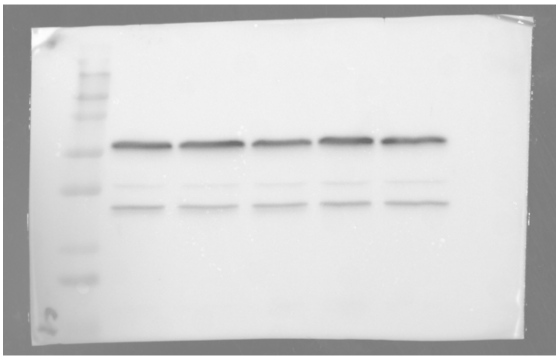

Anti-phospho-eIF2α

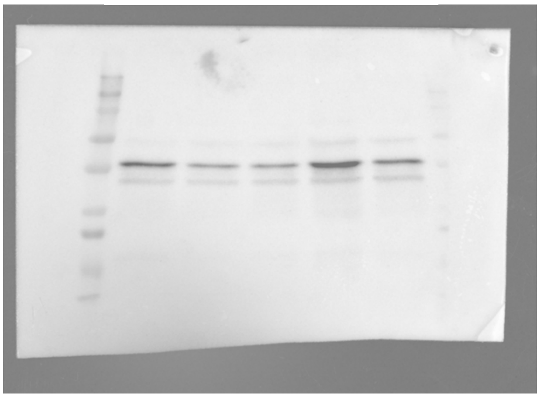

Anti-tubulin

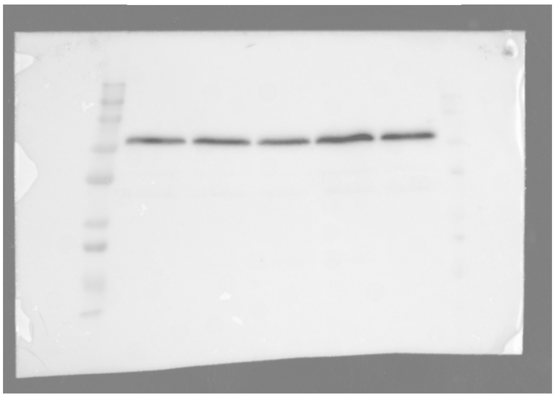

Figure S1d:

Anti-phospho-eIF2α

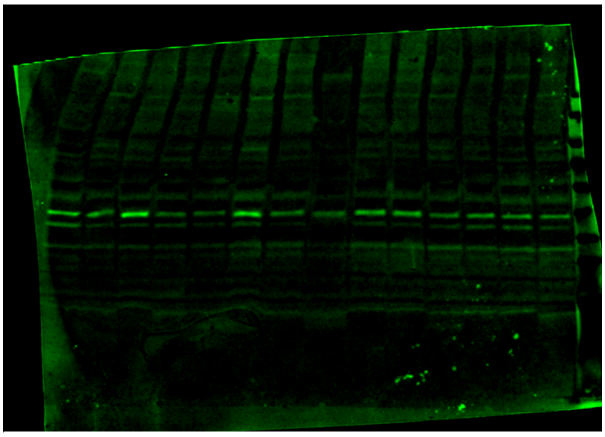

Anti-eIF2α

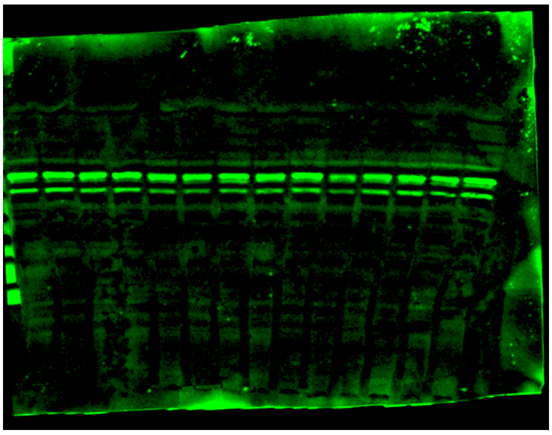

Tubulin

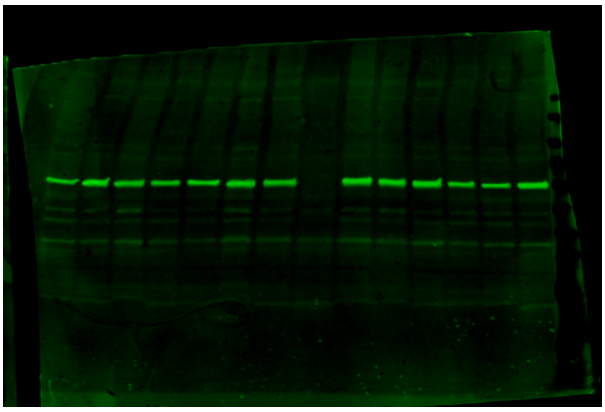

Tubulin

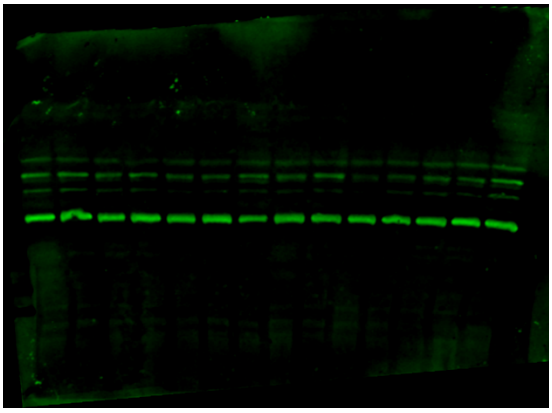

Figure S3b:

ATX2

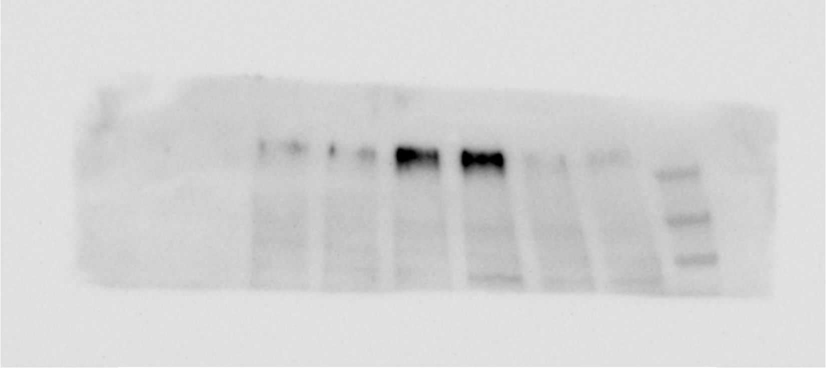

Tubulin

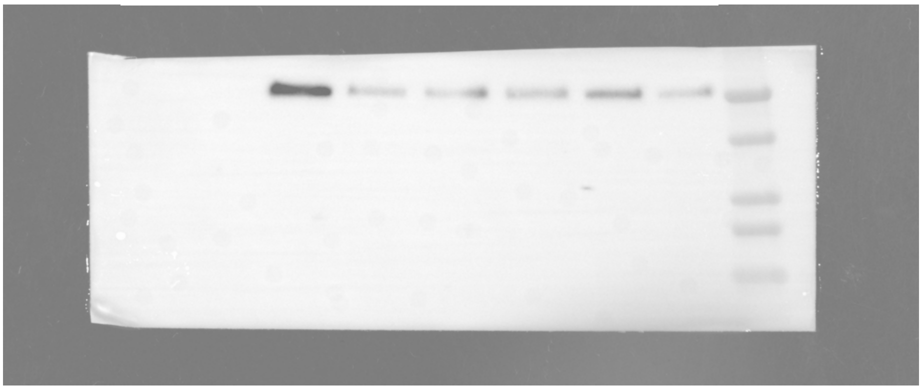

Supplement: Supplementary file 1 — Supplementary Material 1. [file 40478_2026_2301_MOESM1_ESM.pdf]
