# Supplementary material for: Targeting the integrated stress response or Ataxin-2 alleviates neurodegeneration in PolyGR models of C9orf72 associated frontotemporal dementia and amyotrophic lateral sclerosis
Source: Acta Neuropathol Commun. 2026 May 5;14:124. doi: 10.1186/s40478-026-02301-2 (PMC13251143; doi:10.1186/s40478-026-02301-2)
Supplement: Supplementary file 2 — Supplementary Material 2. [file 40478_2026_2301_MOESM2_ESM.docx]

**Fig. S1.**

**a.** QRTPCR quantification of PPP1R15 in the heads of *Drosophila* pan-neuronally (nSyb-Gal4) overexpressing UAS-PPP1R15. Error bars = SEM. Paired t-test.

**b.** QRTPCR quantification of GCN2 in the heads of *Drosophila* pan-neuronally (nSyb-Gal4) expressing UAS-GCN2-RNAi. Error bars = SEM. Paired t-test.

**d.** QRTPCR quantification of PEK in the heads of *Drosophila* pan-neuronally (nSyb-Gal4) expressing UAS-PEK-RNAi. Error bars = SEM. Paired t-test.

**d.** Representative western blots showing levels of eIF2, p-eIF2α and α-tubulin in the heads of *Drosophila* pan-neuronally (nSyb-Gal4) co-expressing GR(1000), or an mCD8-GFP control, with PPP1R15, GCN2-RNAi, PEK-RNAi, PEK^e01744^, ATX2-RNAi^1^ or an mCherry-RNAi control.

**e.** Quantification of the p-eIF2α/eIF2α ratio in the heads of *Drosophila* pan-neuronally (nSyb-Gal4) co-expressing GR(1000), or an mCD8-GFP control, with PPP1R15, GCN2-RNAi, PEK-RNAi, PEK^e01744^, ATX2-RNAi or an mCherry-RNAi control. Error bars = mean ± SEM. ANOVA with post-hoc Šídák’s multiple comparison test, ** p < 0.01, *** p < 0.001, **** p < 0.0001. n = shown on bars

**Fig. S2.**

**a.** Representative micrographs showing eIF4E staining in *Drosophila* primary neurons from flies pan-neuronally (nSyb-Gal4) expressing GR(1000), PR(1000), or an mCD8-GFP control and wild-type primary neurons treated with sodium arsenite (NaAsO_2_). Scale bars = 10 μm.

**b.** Quantification of the percentage of neurons containing eIF4E puncta in primary cultures pan-neuronally (nSyb-Gal4) expressing GR(1000), PR(1000), or an mCD8-GFP control and wild-type primary neurons treated with sodium arsenite (NaAsO_2_). Error bars = SEM, Chi-Squared with Bonferroni correction *** p < 0.001, **** p < 0.0001. n = shown on bars, N = 3.

**c.** Quantification of the number of eIF4E puncta observed in primary cultures from *Drosophila* pan-neuronally (nSyb-Gal4) expressing GR(1000), PR(1000), or an mCD8-GFP control and wild-type primary neurons treated with sodium arsenite (NaAsO_2_). Error bars = SEM, Kruskall-Wallis with post-hoc Dunn’s comparison to controls * p < 0.05, ** p < 0.01, *** p < 0.001, **** p < 0.0001. n = shown on bars, N = 3.

**d.** Representative micrographs showing accumulation of FMR1 in the brains of *Drosophila* pan-neuronally (nSyb-Gal4) expressing 1000-repeat DPRs, or an mCD8-GFP control at 28 days post-eclosion. Scale bars = 20 μm.

**e.** Quantification of the number of FMR1 positive puncta in the brains of *Drosophila* pan-neuronally (nSyb-Gal4) expressing 1000-repeat DPRs, or an mCD8-GFP control at 28 days post-eclosion. Error bars = SEM, ANOVA with post-hoc Dunnett's comparison to controls * p < 0.05.

**f.** Representative micrographs showing accumulation of FMR1 in the brains of wild-type *Drosophila* following heat-stress (2h, 37°C) at 5 days post-eclosion. Scale bars = 20 μm.

**g.** Quantification of the number of FMR1 positive puncta in the brains of wild-type *Drosophila* following heat-stress (2h, 37°C) at 5 days post-eclosion. Error bars = SEM, unpaired T-test ** p < 0.01.

**Fig. S3.**

**a.** Quantification of climbing speed in flies pan-neuronally (nSyb-Gal4) co-expressing GR(1000), or an mCD8-GFP control (Control), with either an RNAi control (UAS-mCherry-RNAi) or UAS-ATX2-RNAi, at 28 Days post-eclosion. Error bars = SEM, Kruskall-Wallis with post-hoc Dunn’s comparison to controls * p < 0.05.

**b.** Representative western blots showing the abundance of ATX2 in the heads of flies pan-neuronally (nSyb-Gal4) co-expressing GR(1000), or an mCD8-GFP control (Control), with two independent ATX2 RNAi lines. 28 days post-eclosion.

**c.** The relative abundance of ATX2 in the heads of flies pan-neuronally (nSyb-Gal4) co-expressing GR(1000), or an mCD8-GFP control (Control), with two independent ATX2 RNAi lines. Normalised to tubulin. 28 days post-eclosion. Error bars = SEM, ANOVA with post-hoc Šidák’s comparison between groups ** p < 0.01, *** p < 0.001.

**d.** Expression of human Ataxin-2 with normal (22Q) and intermediate (32Q) polyglutamine expansions does not modify DPR toxicity when expressed in the *Drosophila* eye.

**e.** Quantification of the percentage of flies pan-neuronally (nSyb-Gal4) expressing 1000-repeat DPRs, or an mCD8-GFP control displaying seizure phenotypes following mechanical (vortex) stimulation at 14 Days post-eclosion. Error bars = SEM, Chi-Squared with Bonferroni correction ** p < 0.01.

**Table. S1. Šídák's multiple comparisons test, 7 vs 21 DPE**

| Genotype |  | Adjusted p value | | | | |
| --- | --- | --- | --- | --- | --- | --- |
|  |  | eIF2α |  | p-eIF2α |  | p-eIF2α:eIF2α ratio |
| Control |  | 0.9997 |  | >0.9999 |  | >0.9999 |
| AP1000 |  | >0.9999 |  | 0.9991 |  | 0.9075 |
| PR1000 |  | 0.9837 |  | 0.8805 |  | >0.9999 |
| GR1000 |  | 0.4885 |  | >0.9999 |  | 0.2628 |
| GA1000 |  | 0.9993 |  | 0.5977 |  | 0.5750 |
